# Rapid Classification and Identification of Multiple Microorganisms with Accurate Statistical Significance via High-Resolution Tandem Mass Spectrometry

**DOI:** 10.1007/s13361-018-1986-y

**Published:** 2018-06-05

**Authors:** Gelio Alves, Guanghui Wang, Aleksey Y. Ogurtsov, Steven K. Drake, Marjan Gucek, David B. Sacks, Yi-Kuo Yu

**Affiliations:** 1 National Center for Biotechnology Information, National Library of Medicine, National Institutes of Health, Bethesda, MD 20894 USA; 2 Proteomics Core, National Heart, Lung, and Blood Institute, National Institutes of Health, Bethesda, MD 20892 USA; 3 Critical Care Medicine Department, Clinical Center, National Institutes of Health, Bethesda, MD 20892 USA; 4 Department of Laboratory Medicine, Clinical Center, National Institutes of Health, Bethesda, MD 20892 USA

**Keywords:** Pathogen identification, Microorganism classification, Statistical significance, Mass, Spectrometry, Proteomics

## Abstract

Rapid and accurate identification and classification of microorganisms is of paramount importance to public health and safety. With the advance of mass spectrometry (MS) technology, the speed of identification can be greatly improved. However, the increasing number of microbes sequenced is complicating correct microbial identification even in a simple sample due to the large number of candidates present. To properly untwine candidate microbes in samples containing one or more microbes, one needs to go beyond apparent morphology or simple “fingerprinting”; to correctly prioritize the candidate microbes, one needs to have accurate statistical significance in microbial identification. We meet these challenges by using peptide-centric representations of microbes to better separate them and by augmenting our earlier analysis method that yields accurate statistical significance. Here, we present an updated analysis workflow that uses tandem MS (MS/MS) spectra for microbial identification or classification. We have demonstrated, using 226 MS/MS publicly available data files (each containing from 2500 to nearly 100,000 MS/MS spectra) and 4000 additional MS/MS data files, that the updated workflow can correctly identify multiple microbes at the genus and often the species level for samples containing more than one microbe. We have also shown that the proposed workflow computes accurate statistical significances, i.e., *E* values for identified peptides and unified *E* values for identified microbes. Our updated analysis workflow MiCId, a freely available software for Microorganism Classification and Identification, is available for download at https://www.ncbi.nlm.nih.gov/CBBresearch/Yu/downloads.html.

**Electronic supplementary material:**

The online version of this article (10.1007/s13361-018-1986-y) contains supplementary material, which is available to authorized users.

**Graphical Abstract:**
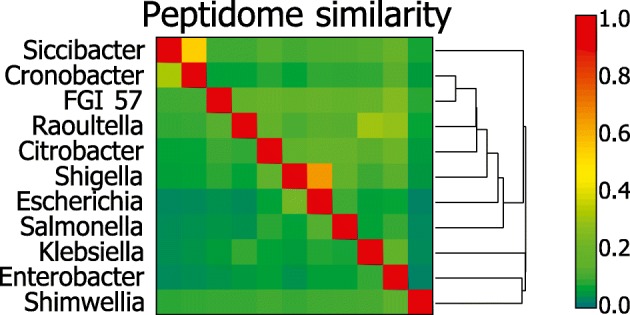
ᅟ

## Introduction

Rapid and accurate identification and classification of microorganisms is of paramount importance to public health and safety [1–3]. Traditional methods for microbial identifications target only a limited number of microorganisms [4, 5] and often require 72 h or more to carry out [6–8]. For most microorganismal identification protocols, the first step can take the longest. This time-consuming step involves preparing a culture of the collected sample in a selected medium, usually a blood culture, to test for the presence of any microbes and to amplify the concentration of microbes that might be present [7–9]. If the prepared culture tests positive for the presence of microbes, further tests are required to distinguish and identify within the sample each microbe present [7, 8, 10, 11].

One of these tests is the analytical profile index (API), which consists of a system of 20 biochemical reactions. Among the concerns with the API test are that it cannot always identify microbes at the species level and it cannot handle samples composed of multiple microbes [12]. Another frequently used test is the enzyme-linked immunosorbent assay (ELISA), a highly specific yet expensive test, which relies on the use of antibodies that are specific to antigens of a given species of microbe [13, 14]. Traditional polymerase chain reactions (PCR) of 16S rRNA followed by sequencing methods [15] are also utilized in the identification of microbes. One issue in using 16S rRNA sequence information is that it provides reliable identification at the genus level for the majority of cases, but it cannot always be used to differentiate between closely related species having a 16S rRNA sequence similarity greater than 97% [16, 17]. For example, *Bacillus globisporus* and *Bacillus psychrophilus* have greater than 99.5% sequence homology in their 16S rRNAs but have a DNA-level relatedness of only 23–50% when measured by hybridization reaction [18, 19]. Studies using FilmArray multiplex PCR have been shown to be able to early detect single or multiple pathogenic microbes present in positive blood cultures, as long as pathogenic microbes are present with high enough concentrations and the FilmArray system has the proper primers for these pathogenic microbes [20]. There are also methods that do not require a blood culture and can identify microbes from whole blood samples. Of these methods, PCR amplification for electrospray ionization mass spectrometry (PCR/ESI-MS) is the most promising with reported sensitivity and specificity in the 1990s [8].

In recent years, next-generation sequencing (NGS) and mass spectrometry (MS) have emerged as reliable technologies for rapid and accurate identification/classification of microbes [21, 22]. Utilizing both the coding and noncoding DNA information, NGS can be used to screen bacteria effectively although it lacks gene expression information. On the other hand, the MS-based technology (the focus of the current manuscript) identifies microbes via peptides found thus providing protein expression information. There are different ways in which these technologies can be employed for microorganismal identification, and we direct the reader to the review articles of Hodkinson and Grice [23] and of Saurce and Klien [22] for a survey.

Both the NGS and MS-based technologies can be routinely used to identify single microbes. For example, matrix-assisted laser desorption ionization time-of-flight MS (MALDI-TOF-MS) [24, 25] can be employed to identify single microbes quickly and accurately in pure samples [26]. However, it cannot be used for identification in a sample containing multiple microbes [27, 28]. Given that our main goal is to robustly and accurately identify multiple microbes in mixed samples, in our analyses, we use only data collected from high-resolution instruments using high-performance liquid chromatography-tandem MS (LC-MS/MS) [29] to mitigate one of the challenges (noise in data) that hinders the identification of multiple microbes. Another challenge appears due to the fast expansion of database size. In order not to miss identify any known microbe, one needs as an input to peptide identification tools a protein database that includes all microbes present in the sample. The ever-increasing number of microbial proteomes implies an ever-expanding peptide/protein database along with ever-increasing number of post-translational modifications (PTMs) as well as single amino acid polymorphisms (SAPs) that will overwhelm most peptide identification tools [30].

In addition to having longer search time, the consequence of using a much larger database includes reduction of sensitivity. One way to circumvent this is to first construct a peptide-centric database without including PTMs or SAPs, sorted according to peptide masses, then have an interface program that extracts from the database, for every precursor ion mass of the query spectrum, the corresponding peptide set and passes it to peptide identification tools as the input database. PTMs and SAPs are then allowed only for microbes whose identification confidences are higher than a specified threshold. Although this strategy allows for a higher peptide identification rate, it should not be confused with the multistage proteomics search strategies based on the target-decoy statistics [31] that is often used in the metaproteomics community. For this type of multistage search strategy, in the second step (re-search), the resulting proportion of false discoveries (or false discovery rate) can no longer be estimated correctly [32–34], hence undermining their validity. When using methods that do not require target-decoy approach to assign accurate statistical significance, however, the difference between the multistage and one-pass search strategies becomes that of using a smaller database and a larger one. And the sensitivity advantage of multistage strategies can be achieved with much less search time by the stratified database search method [35]. However, even if the database size issue can be mitigated by using stratified database and on-the-fly scope expansion of PTMs and SAPs, a major challenge remains in terms of identifying multiple microbes.

This major challenge originates from the difficulty in delineating the microbes based on peptides identified. We meet this challenge by developing a method that can delineate or group microbes based on their peptidome similarity (counting only experimentally observed peptides of high confidence) and can assign accurate statistical significances to microbes identified. In terms of identifying multiple microbes, the aforementioned challenges are common to both the NGS [36–38] and MS-based methods [39–45]. However, the extent to which one can apply the statistical methods developed in this manuscript to NGS-based methods deserves a separate investigation that is beyond the scope of the current manuscript. In addition to the two main challenges outlined above, existing MS-based methods for microbial identification/classification [39–44] as mentioned by Boulund et al. [45] also face other challenges such as being limited to simple samples and needing manual intervention during data analysis. The latter makes it hard to automate the data analysis workflow.

Interestingly, identifying multiple microbes is also pursued in areas such as environmental proteomics, also termed metaproteomics [46–48]. In this area, even though the primary aim is to understand the functional expression of complex samples (not the microbe classification/identification in particular), recently the reliability of taxonomic attribution using the bioinformatic tools Unipept [49] and MEGAN [50] has been assessed using unique (taxon-specific) peptides found experimentally [51]. This latter endeavor is equivalent to classification/identification of microbes [47, 48], which happens to be our main goal. However, unlike Unipept [49] and MEGAN [50], our method can identify/classify microbes with accurate statistical significance assignment. Our method can also be applied in terms of functional expression. To achieve this, we identify the peptides, then the microbes, and then the corresponding proteins. With proteins identified, one may query the term databases such as gene ontology to obtain functional expression [52].

In this manuscript, we present an updated version of MiCId, an analysis workflow for rapid and accurate identifications/classifications of microbes. MiCId was designed to automate the complete process, from microbial peptide database construction to microbial identification and protein identification. The first version of MiCId, standing for Microorganism Classification and Identification, was tested for analysis of samples containing a single microbe [53]. We have now updated MiCId to specifically handle mixed samples containing multiple microbes while preserving its speed and its accurate statistical significance. Using 226 LC-MS/MS data files from a variety of microbial samples, some of whose microbial compositions are unknown, and 4000 blended MS/MS data files, we have extensively evaluated MiCId’s performance in terms of identifying/classifying multiple microbes at different taxonomic levels. MiCId utilizes a hierarchical identification strategy where microbes are identified starting at the phylum level then descending one level at a time. With the *E* value cutoff set at 0.01 (effectively control the PFD to be less than 5%), in terms of microbe identification using blended MS/MS data (BMD-A and BMD-B), MiCId yields an average true positive rate of 0.9813 at the genus level and 0.9550 at the species level. (More details can be found in Table 2.) One should note, however, that these numbers were obtained by blending spectra from up to 24 single-species samples. Generalization to complex microbiota samples should be taken with a grain of salt. MiCId’s computed statistical significance is shown to be accurate for microbes identified when tested against a decoy database at various taxonomical levels, providing a confidence measure for users to control the proportion of false discoveries. The proposed workflow has been implemented in MiCId, a freely available software that can be downloaded at http://www.ncbi.nlm.nih.gov/CBBresearch/Yu/downloads.html.

## Materials and Methods

### Downloaded MS/MS Data Files

A total of 207 LC-MS/MS data files were downloaded from the ProteomeXchange database at http://www.proteomexchange.org/ and from PeptideAtlas at http://www.peptideatlas.org/. Of these LC-MS/MS data files, 194 are from mixture samples of known organisms, with each sample containing one, two, four or nine organisms. The remaining 13 data files are from complex samples of the human gut. Supplementary Tables S1, S2, S3, and S4 provide the data file (DF) number, the file name, and the ProteomeXchange or PeptideAtlas identifier for each LC-MS/MS data file downloaded. All the MS/MS spectra described here were acquired on high-resolution mass spectrometers and further experimental details can be obtained in the ProteomeXchange website. The true positive microbes in the latter group of data (human gut microbiome) are unknown. For the data from samples in the former group, the true positives in each sample were provided along with their mix ratios; many such samples have nearly equal number of cells per microbe, and some are from very biased microbe populations. To complement the ratio varieties of the downloaded data, we also generated some in-house MS/MS data from samples with more biased (but not extreme) ratios.

### In-House Dataset

#### Bacterial Culture Preparation

Fresh *Escherichia coli* (ATCC 25922), *Pseudomonas aeruginosa* (ATCC 27853), *Streptococcus pneumoniae* (ATCC 49619), and *Staphylococcus aureus* (ATCC 25923) plates were used to inoculate a 2 ml tryptic broth for overnight growth. From each saturated culture, a 10-ml vial was inoculated with 100 μl (1:100 dilution) and put in shaker at 37 ^°^C. Each culture growth was monitored by nephelometer to an optical density value between 0.4 and 0.51 (approximately 10^9^ cells). True CFU values were achieved by plating diluted samples on sheep blood agar plates and counting the resulting colonies. Based on the measured approximate cell count values for each culture, these cultures were mixed in different ratios to generate microbe mixtures containing 10^9^ cells total. The prepared microbe mixtures with their estimated ratios, DFs 84–102, are listed in Table S3. These mixtures were added to eppendorf tubes and spun at 14 K rpm for 2 min until all of the samples were in the eppendorf tube and the supernatants discarded. These pellets were washed with 1 ml 70% EtOH and then resuspended in 150 μl 70% formic acid. After vortexing, 150 μl acetonitrile was added and samples were vortexed and respun. The supernatant was transferred to a clean tube and speed-vacuum dried on medium heat. To each dried tube, 40 μl of 6 M urea and 50 mM NH_4_HCO_3_ were added and the tube was sonicated for 50 min with occasional vortexing. Samples were reduced with DTT (4 μl 1 M in water, 37 °C for 60 min), alkylated (20 μl iodoacetamide 40 mg/ml in water, at room temperature for 60 min in the dark), and quenched with DTT (4 μl, 15 min). The tubes were diluted with 100 μl of 50 mM NH_4_HCO_3_ and 10 μl of 100 mM NH_4_HCO_3_. Trypsin/Lys-c (Promega, 2 μg) was added to each tube. Samples were digested using the CEM Discovery microwave digester (60 min, 50 °C, 50 W, with cooling). After digestion, samples were stored at − 20 °C until used.

#### Liquid Chromatography-Tandem Mass Spectrometry Acquisition

Liquid chromatography-tandem mass spectrometry (LC-MS-MS) was performed using an Eksigent nanoLC-Ultra 2D system (Dublin, CA) coupled to an Orbitrap Elite mass spectrometer (Thermo Scientific, San Jose, CA). Peptide samples were first loaded onto a Zorbax 300SB-C18 trap column (Agilent, Palo Alto, CA) at a flow rate of 6 μL/min for 10 min, and then separated on a reversed-phase BetaBasic C18 PicoFrit analytical column (0.075 × 250 mm, New Objective, Woburn, MA) using a 90-min linear gradient of 5–35% acetonitrile in 0.1% formic acid at a flow rate of 250 nl/min. Eluted peptides were sprayed into the Orbitrap Elite equipped with a nano-spray ionization source. Both survey (MS) and product (MS/MS) spectra were acquired in the Orbitrap, and the FTMS resolution was set at 30,000 and 15,000, respectively. Each MS scan was followed by six data-dependent CID MS/MS scans with dynamic exclusion. Other mass spectrometry settings were as follows: spray voltage, 1.8 kV; full MS mass range, *m*/*z* 300 to 2000; normalized collision energy, 35%; precursor ion isolation mass width, 3 Da.

### Blended MS/MS Dataset

Even though we already have some data files from mixture samples of up to nine microbes, they are limited in number and perhaps in complexity. While we have the data files from the complex samples of the human gut, the true positives within these samples are unknown. To stress test our proposed identification method, we need a large dataset made of highly complex samples but with true positives known. Absent existing DFs of this type, we generated blended DFs in silico (similar to methods of [54] that have been employed to evaluate metagenomics analysis workflows).

Each blended MS/MS data file for this purpose was generated using the following steps: (1) identify a list of data files, each containing MS/MS spectra from a sample of a microbe; (2) for each data file in the list, a number of MS/MS spectra out of the total were randomly sampled according to a pre-specified percentage; (3) merge the sampled MS/MS spectra to mimic a data file from a mixture sample of these microbes; and (4) repeat steps 1–3 to achieve the desired number of blended MS/MS data files. The number of microbes chosen and the specified percentage of MS/MS spectra to sample (from each microbe’s MS/MS data file) determine the size of a blended MS/MS data file.

A total of three blended MS/MS datasets (BMDs) were generated. BMD-A was used for learning the parameters needed for MiCId’s clustering procedure; BMD-B was used to evaluate MiCId’s performance in terms of sensitivity and specificity; BMD-C was used to evaluate the accuracy of MiCId’s statistical significance assignment in terms of microbe identifications.

DFs 1–24, which cover 24 species and 21 genera, were used to generate BMD-A. BMD-A is composed of five subsets, each corresponding to a fixed sampling percentage of 1, 5, 10, 25, or 50%. Every subset contains 500 blended DFs, each of which was generated by sampling a fixed percentage of MS/MS spectra from every one of the 24 DFs. BMD-B, covering 15 species and 15 genera, is made of seven subsets each composing 200 blended DFs. Every blended DF in a subset was generated by sampling a fixed percentage *p* of MS/MS spectra from group 1 DFs and a complement percentage *100-p* of MS/MS spectra from group 2 DFs. For BMD-B, group 1 contains DFs 24–31 and group 2 contains DFs 32–38. The seven different subsets are distinguished by their complement pairs of percentages: (95, 5%), (90, 10%), (75, 25%), (50, 50%), (25, 75%), (10, 90%), (5, 95%). BMD-C contains 100 blended DFs. Every blended DF of BMD-C was generated by sampling 50% of MS/MS spectra from each of the following DFs: 40, 43, 48, 53, 55, and 61.

### Peptide-Centric Databases

Protein sequences were downloaded (on February 16, 2018) from the National Center for Biotechnology Information (NCBI) at ftp://ftp.ncbi.nlm.nih.gov/genomes/genbank/. For the current study, five peptide-centric databases (DBs) (DB-1 through DB-5) were constructed. In the genbank.assembly file, an organism’s genome assembly level is labeled as contig, scaffold, chromosome, or complete genome, in order of increasing completeness. The peptide-centric DB-1 (DB-2) includes, in addition to *Homo sapiens* and *Mus musculus*, all bacteria, archaea, fungi, and virus whose assemblies are at chromosome (scaffold) level or higher. The other three databases were created from metagenomics data that have been analyzed and transformed into protein sequence fasta files [55]: DB-3 (DB-Human_0_RF_6GB.fasta containing 3,423,708 sequences), DB-4 (DB-Human_0_CF_6GB.fasta containing 192,582 sequences), and DB-5 (DB-Human_1+2+3_RF_6GB.fasta containing 2,866,541 sequences).

All DBs were constructed as follows: downloaded protein sequences were in silico digested following the digestion rule for trypsin and lys-c, i.e., cleaving at the carboxyl termini of arginine and lysine, allowing up to two missed cleavage sites. In our DBs, only *nonredundant* tryptic and lys-c peptides with molecular masses between 660 and 4000 Da were kept. By nonredundant peptide, we mean the following. We keep a copy and only one copy of *every* possible peptide (resulted from in silico enzyme digestion of the protein database) regardless of whether the peptide is shared by multiple microbes or not. A nonredundant peptide therefore can be a unique peptide to a microbe at a given taxonomic level, but may become a shared peptide when a lower taxonomic level is considered. Nevertheless, no peptides will be excluded, and as more microbe genomes are sequenced, the growth rate of our peptide-centric database is expected to be smaller than that of the protein databases.

In DB-1 and DB-2, for each peptide, the names of strains, subspecies, species, genera, families, orders, classes, and phyla that contain this peptide are also recorded and linked to the peptide. The sizes of DB-1 and DB-2 are 46 and 200 GB, respectively. Taxonomic information included in DB-1 and DB-2 was extracted from the taxonomy files downloaded (on February 16, 2018) from the NCBI (https://www.ncbi.nlm.nih.gov/Taxonomy). The 46,838,064 protein sequences (807,574,956 nonredundant tryptic peptides) in DB-1 are from 23,911 organisms, belonging to 13,072 species, 1890 genera, and 517 families. The 260,931,852 protein sequences (2,507,889,685 nonredundant tryptic peptides) in DB-2 are from 75,356 organisms, belonging to 26,368 species, 2870 genera, and 701 families. Relevant information pertaining to the NCBI taxonomy identifiers and organism names for the different organisms included in DB-1 and DB-2 can be found in Supplementary Table S5. Since DB-3, DB-4, and DB-5 are from metagenomics reads/contigs, no taxonomy information is available.

### Software Parameter Values Used

Six databases, DB-1 through DB-5 and the reverse of DB-1, were used in MS/MS data analyses; the first five were used as the target DBs while the last as the decoy DB. The other software parameters are described below. While performing database searches, the digestion rules of trypsin and lys-c were assumed with up to two missed cleavage sites per peptide allowed. Iodocetamide was used as the reduction agent, changing the molecular mass of every cysteine from 103.00919 to 160.030647 Da. The mass error tolerance of 10 ppm was set for both precursor and product ions. RAId’s Rscore scoring function, using b and y ions as evidence, was used for scoring peptides. The statistical significance assigned to each peptide was given by RAId’s theoretically derived peptide score distribution [56]. The largest (cutoff) *E* value for a peptide to be reported was set to 1.

### Statistical Method for Microbial Identification

For our statistical method for microbial identification to be effective, two prerequisites are indispensable: (1) accurate significance assignments, e.g., *E* values, at the peptide level must be provided and (2) microbes used for database construction must have the correct taxonomic classification. The first requirement is satisfied because peptide identifications in MiCId are done by using RAId’s scoring function and significance assignments which have been shown to yield accurate *E* values [56, 57].

As for the second requirement, it is known that the microbes’ taxonomic classification is not perfect and sometimes controversial. For example, some studies recommend that *Shigella flexneri* should be classified as a strain of *Escherichia coli* [58, 59]. However, the microbes’ taxonomic classification is expected to improve thanks to recent advances in DNA sequencing technology and a polyphasic approach that utilizes genotypic, chemotypic, and phenotypic information during taxonomic classification [60].

To provide microbe identification significances, we compute a unified *E* value (*E*_*u*_) by combining the spectrum-specific *E* values of the confidently identified peptides (CIPs). A peptide is considered a CIP if it is identified with an *E* value smaller than a cutoff (*E*_*c*_). When performing identifications, we might want to eliminate potential false positives aggressively thus setting *E*_*c*_ low; currently, *E*_*c*_ is defined to be the minimum of 1 and 100/*n*_*s*_ (with *n*_*s*_ denoting the total number of MS/MS spectra acquired for a given experiment). (When assessing statistical accuracy using a random/decoy database, however, one is essentially counting false positives and setting *E*_*c*_ too low will reduce the number of false positives estimated.) With this *E*_*c*_ specified, on average for large *n*_*s*_, only 100 false positive peptides are expected among the CIPs. We next detail an important clustering step [53], which is now improved to accommodate identifications of multiple microbes.

In our peptide-driven clustering procedure [53], taxa sharing significant amounts of CIPs were clustered together. All CIPs were regarded as equally important, and the clustering procedure did not go through further iteration. In this manuscript, we incorporate all peptides with *E* values less than 1 in the clustering procedure; however, we also introduce fractional counts to give peptides with better (lower) *E* values more (less) influence than peptides with worse (higher) *E* values. The updated peptide-driven clustering procedure is described below.

First, peptides identified with *E* values ≤ 1 are mapped to the different taxa in the database. Second, a standardized weighted count (*Z*) is assigned to each identified peptide (whose *E* value is *E*).

1 $$ Z(E)=\frac{1}{\left(1+\frac{E}{E_c}\right)}. $$

Third, taxa are sorted in decreasing order of their weighted number of identified peptides to prepare for the clustering procedure. The first taxon entering a cluster is called the head of that taxon cluster, while other taxa the members of that cluster. Starting from the best ranked taxon (a cluster head) in the sorted list, any other lower ranked taxon will cluster to the former if a resemblance coefficient of 0.85 or larger is obtained between them. The resemblance coefficient is defined as the proportion of the weighted number of peptides belonging to a lower ranked taxon that can be explained by identified peptides associated with a cluster head. Once the worst ranked taxon is reached, the process will continue with the best ranked, not-yet-clustered taxon as a cluster head and repeat until all the unclustered taxa have been attempted as a cluster head, but not more than once.

Fourth, after having generated taxon clusters, we further group these taxon clusters as follows. Starting with the lowest ranking taxon cluster head, we denote its resemblance coefficient to others by its proportion of the weighted number of identified peptides that are shared by all other heads of taxon clusters; when a resemblance coefficient of 0.85 or larger is obtained, the cluster under consideration is merged to its closest taxon cluster, i.e., the one whose head shares the largest weighted number of peptides with the current head. The remaining number of cluster heads *n*_*c*_ is used as the Bonferroni correction factor for significance calculation later. There is, however, an exception to the general clustering rules above. When a taxon (taxon cluster) contains three or more CIPs that are not shared with any other taxa (taxon clusters), it can only be a cluster head.

There is also a difference in the identification workflow compared to our earlier method. We considered all genera (species) together when performing genus (species) level identifications [53]. Here, we begin identifications at the phylum level and then down. The current method has the advantage that one may eliminate from consideration taxa that are unlikely to be present during the upper level identifications. Below is the condition we employ to select taxa to be retained at each identification level. Each cluster head will be considered; any member with a percentage difference in the weighted number of CIPs to its cluster head less than 15% or having three or more CIPs that are not shared with other taxa will be retained.

In order to provide statistical significances at various taxonomic levels, we compute a unified *E* value *E*_*u*_ by combining the spectrum-specific *E* values of the CIPs belonging to the same taxon. The details of how the *E*_*u*_s are computed for microbes at different taxonomic levels have been previously described [53]. Here, we only briefly outline the essential steps for computing *E*_*u*_.

The unified *E* value *E*_*u*_ is given by

2 $$ {E}_u={n}_c\times {P}_u, $$

where in Eq. (2), the unified *P* value (*P*_*u*_) is multiplied by the Bonferroni correction factor, *n*_*c*_, the final number of peptide-driven taxon clusters. The *P*_*u*_ is obtained by first transforming the *E* values (*E*) of CIPs into database *P* values (*p*) [61, 62], *p* = 1 − *e*^−*E*^. A weight (*w*_*π*_) is then defined for each peptide *π* as 1/*C*_*π*_! with *C*_*π*_ being the total number of taxon clusters containing *π*. Note that *w*_*π*_s are computed for all *π*s identified with *E* values ≤ 1 although only CIPs are used for computing the unified *P* value. Evidently, *C*_*π*_ varies by the taxonomic level considered.

For each taxon *T*, one then combines the weighted product of database *P* values into a new variable

3 $$ \tau =\prod \limits_{i=1}^{n_T}{p}_i^{w_i}, $$

where *n*_*T*_ is the total number of CIPs mappable to taxon *T*, and the weight for peptide *π*_*i*_ is ${w}_i=1/{C}_{\pi_i}!$. The sum of peptide weights ${m}_{\mathrm{raw}}\equiv {\sum}_{i=1}^{n_T}{w}_i\left(E\le {E}_c\right)$ gives the effective number of degrees of freedom. This allows one to define a stochastic variable $\overset{\sim }{\tau }$ of the same number of degrees of freedom to compare with, leading to the conditional probability formula for the product of truncated *P* values [53, 63].

4 $$ {P}_t\left(\tilde{\tau}\le \tau |\ m,{m}_{\mathrm{raw}}\right)=\frac{\tau }{P_c^{m_{raw}}}\sum \limits_{s=0}^{m-1}\frac{\left[{m}_{raw}\mathit{\ln}\ \Big({P}_c\right)-\mathit{\ln}\ \left(\tau \right)\Big]{}^s}{s!}\kern0.5em , $$

where *m* ≡ ⌈*m*_raw_⌉ is the smallest integer that is greater than or equal to *m*_raw_. An example of how to compute the unified *P* value using Eq. (5) can be found in the electronic supplementary material of an earlier publication [53].

Finally, *P*_*u*_ is given by

5 $$ {\displaystyle \begin{array}{l}{P}_u\left(\tilde{\tau}\le \tau \right)=\frac{M!}{m!\left(M-m\right)!}{P}_c^m{\left(1-{P}_c\right)}^{M-m}{P}_t\left(\tilde{\tau}\le \tau |\ m,{m}_{\mathrm{raw}}\right)\\ {}\kern2.999999em +\sum \limits_{j=m+1}^M\kern0.5em \frac{M!}{j!\left(M-j\right)!}{P}_c^j{\left(1-{P}_c\right)}^{M-j}\times \\ {}\kern3.719998em \times \left[\theta \left({P}_c^j-\tau \right)\ {P}_t\left(\tilde{\tau}\le \tau |\ j,j\right)+\theta \left(\tau -{P}_c^j\right)\ \right],\end{array}} $$

where $M=\left\lceil {\sum}_{j=1}^{N_T}{w}_j\left(E\le 1\right)\right\rceil$ with *N*_*T*_ being the total number of identified peptides (with *E* value ≤ 1) mappable to taxon *T*, *P*_*c*_ is the database *P* value for *E*_*c*_, and the *θ*(*x*) function takes the value 1 when *x* > 0 and 0 otherwise.

Within each cluster, the unified *E* value of each member taxon, cluster head included, is computed; the taxon with the most significant unified *E* value becomes the head of the cluster. Note that the starting cluster head (with largest *M*) remains most significant most of the time, indicating the stability of our clustering procedure and significance assignment. After this step, the clusters are finally sorted in ascending order of the unified *E* values of the cluster heads.

### Statistical Method for Protein Identification

In this update of MiCId, we have included the protein identification capability. Proteomes of confidently identified microbes (cluster heads at the species level) are used as the protein database. The implemented statistical framework for protein identification is found on a rigorously derived general formula [64], which has been extensively tested for the application to protein identification [65]. Since the detailed derivations [64] and the applications [65] are already available, here we only briefly summarize the statistical method for protein identification in MiCId.

As before, we consider all identified peptides with *E* values *E* ≤ 1 and convert each *E* value of an identified peptide into a database *P* values (*P* = 1 − *e*^−*E*^) [61, 62]. These identified peptides are then mapped to database proteins that contain them. Assume that a given protein contains *L* identified peptides with *P* values. Let us group these *L* peptides, according to the number of database proteins a peptide maps to, into *m* groups with 1 ≤ *m* ≤ *L*. Within each group *k*, the *n*_*k*_ peptide *P* values have equal weight, while peptide *P* values in different groups are weighted differently.

The weighting enters our formalism through the following quantities of interest

6 $$ \tau \equiv \prod \limits_{k=1}^m{\left[\prod \limits_{j=1}^{n_k}{p}_{k;j}\right]}^{w_k}\kern0.5em \mathrm{and}\kern0.5em Q\equiv \prod \limits_{k=1}^m{\left[\prod \limits_{j=1}^{n_k}{x}_{k;j}\right]}^{w_k}, $$

where each *p*_*k*; *j*_ represents a reported peptide database *P* value and each *x*_*k*; *j*_ represents a random variable drawn from a uniform, independent distribution over [0, 1]. The quantity of interest *F*(*τ*) ≡ Prob(Q ≤ *τ*), representing the protein *P* value, can be obtained [64, 65].

7 $$ {\displaystyle \begin{array}{l}F\left(\tau \right)=\left[\prod \limits_{l=1}^m{r}_l^{n_l}\right]\sum \limits_{k=1}^m\ \sum \limits_{\mathcal{G}(k)}\kern0.5em \frac{1}{r_k^{g_k+1}}H\left(-{r}_k\ln\ \tau, {g}_k\right)\times \\ {}\kern0.84em \times \left.\left(\prod \limits_{j=1,j\ne k}^m\frac{\left({n}_j-1+{g}_j\right)!}{\left({n}_j-1\right)!{g}_j!}\frac{{\left(-1\right)}^{g_j}}{{\left({r}_j-{r}_k\right)}^{n_j+{g}_j}}\right)\right\},\end{array}} $$

where *r*_*k*_ ≡ 1/*w*_*k*_ is the number of proteins a group *k* peptide maps to, ${\sum}_{\mathcal{G}(k)}$ enumerates each set of nonnegative integers {*g*_1_, *g*_2_, …, *g*_*m*_} that satisfies the *k*-dependent constraint ${\sum}_{i=1}^m{g}_i={n}_k-1$, and the function *H* is defined as

8 $$ H\left(x,n\right)\equiv \kern0.5em {e}^{-x}\ \sum \limits_{k=0}^n\frac{x^k}{k!}\kern0.5em . $$

An example application of Eq. (7) can be found in the supplementary information of the published study on protein identification [65].

## Results and Discussion

**Figure 1:**
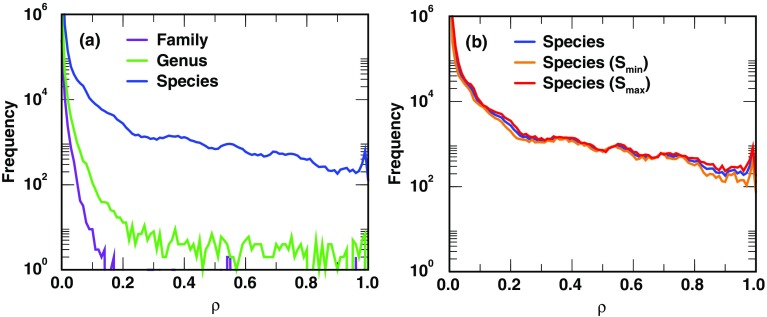
Histograms of microbial peptidome similarity (*ρ*). The curves display the peptidome similarity histograms in DB-1. In general, asymmetric peptidome similarity is used: similarity of taxon *X* to *Y*, *S*_*X* → *Y*_, is defined by *S*_*X* → *Y*_ ≡  ∣ *X* ∩ *Y* ∣ / ∣ *X*∣, the number of shared peptides between the two taxa divided by the number of peptides corresponding to taxon *X*. Note that both *S*_*X* → *Y*_ and *S*_*Y* → *X*_ are included in the histogram. To illustrate that the high frequency of large peptidome similarity among species is generic, we defined in addition two symmetrized similarities: *S*_max_(*X* → *Y*) = *S*_max_(*Y* → *X*) ≡  ∣ *X* ∩ *Y* ∣ / min {| *X*| , | *Y*| } and *S*_min_(*X* → *Y*) = *S*_min_(*Y* → *X*) ≡  ∣ *X* ∩ *Y* ∣ / max {| *X*| , | *Y*| }. These new symmetrized measures are shown only for peptidome similarities among species. The closeness among all three versions of similarity measures for species indicates that the presence of high peptidome similarity among species is generic, not a consequence of asymmetric similarity

To investigate the feasibility of microbial identification based on peptides identified, we examine in silico the peptidome similarities among microbes at different taxonomic level in our DB-1. The similarity of taxon *X* to *Y*, *S*_*X* → *Y*_ ≡  ∣ *X* ∩ *Y* ∣ / ∣ *X*∣, is defined as the numbered of shared peptides between the two taxa divided by the number of peptides corresponding to taxon *X*. Panel a of Figure 1 displays histograms of the in silico peptidome similarities computed among the families, genera, and species in DB-1. The histograms in Figure 1 show that peptides are weakly shared among taxa above species level having similarity values that are typically much less than 0.6, indicating that microbial identifications at levels higher than species should be easier than at the species level. Based on these histograms, it appears that unambiguous identification at the species level for certain species can be challenging. To investigate whether the high peptidome similarity at the species level is an artifact due to asymmetric similarity measure, we defined in addition two symmetrized similarities: *S*_max_(*X* → *Y*) = *S*_max_(*Y* → *X*) ≡  ∣ *X* ∩ *Y* ∣ / min {| *X*| , | *Y*| } and *S*_min_(*X* → *Y*) = *S*_min_(*Y* → *X*) ≡  ∣ *X* ∩ *Y* ∣ / max {| *X*| , | *Y*| } and plot the species-level peptidome similarity histograms using all three similarity measures (see panel b of Figure 1). The closeness among all three versions of similarity measures for species indicates that the presence of high peptidome similarity among species is generic, not a consequence of asymmetric similarity. However, similar to the well-known example of high peptidome similarity between *E. coli* and *S. flexneri* [58, 59], the exceedingly high peptidome similarities observed among certain species may also be a consequence of incorrect/problematic taxonomy classifications.

The question of what to include/exclude from the database is definitely important, and the answer probably varies depending on the research conducted. It has been shown that the choice of databases affects microbial identification [55]. As another example, researchers studying the human gut microbiome may wish to construct a gut-specific database [48] by including only human gut microbial species that have been cataloged by the Human Microbiome Project [66]. To facilitate microbial research of various types, MiCId offers a simple procedure for constructing a customized database and using it for microbial identification afterwards. A user only needs to specify a list with the names of species or their NCBI taxonomy identifier, and everything else is handled by MiCId.

### *E* Value Accuracy Evaluation

As stated in the “Materials and Methods” section, the statistical method employed to assign statistical significance to microbes identified requires accurate per-spectrum significances, e.g., *E* values, at the peptide level. Combining RAId’s accurate per-spectrum statistics [56] to output unified *E* value (*E*_*u*_) for microbes identified, we expect *E*_*u*_ to be accurate and evaluated its accuracy as described below. MS/MS spectra from BMD-C were used to query the reverse [67, 68] of DB-1 while keeping the taxonomic assignments untouched. The reverse of DB-1 thus acts as a peptide database from decoy organisms. (Therefore, each decoy organism carries a canonical name, but its peptide sequences are reverse of those of the true organism of the same name.) The unified *E* value *E*_*u*_ of each *decoy* organism can then be calculated using Eqs. (2) and (5). Obviously, each identified decoy organism is a false positive. The accuracy of assigned statistical significance (or type I error) can be visualized by a log-log plot [69, 70] of the expected number of errors (decoy organisms identified) per query versus the *E*_*u*_ reported by MiCId.

**Figure 2:**
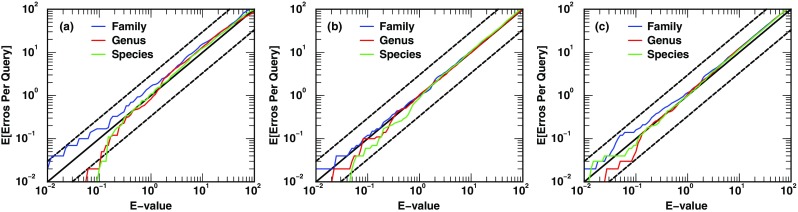
Accuracy assessment of the unified *E* value (*E*_*u*_) for microorganism identifications. Each of the 100 blended DFs in BMD-C was queried in the decoy (made of reversed target peptide sequences) of the target DB-1. Three *E*_*c*_s, peptide *E* value cutoffs, are used: (**a**) *E*_*c*_ = 0.01, (**b**) *E*_*c*_ = 0.1, and (**c**) *E*_*c*_ = 0.5. The closer the curve is to the *y* = *x* line, the more accurate the reported *E*_*u*_

Since we have in total 100 queries (blended DFs) in BMD-C, one would expect 1 decoy microbial cluster head with *E* ≤ 10^−2^, 10 decoy microbial cluster heads with *E* ≤ 10^−1^, 100 microbial cluster heads with *E* ≤ 10^0^, and 10,000 microbial cluster heads with *E* ≤ 10^2^. The log-log plot of the expected number of errors per query versus *E*_*u*_ should yield a curve close to the *y* = *x* line. Panels a, b, and c of Figure 2 show that the computed *E*_*u*_ curves trace very closely the *y* = *x* line, indicating that the computed *E*_*u*_ is indeed accurate. Two dashed lines, *y* = 3*x* and *y* = *x*/3, are also provided as references. As described in the “Statistical Method for Microbial Identification” subsection, when assessing the accuracy of assigned significance using decoy databases, one should not set *E*_*c*_ too low and the statistical accuracy should remain even with different *E*_*c*_. To test this, we have used three *E*_*c*_ values, 0.01, 0.1, and 0.5, corresponding to curves in panels a, b, and c, respectively. These *E*_*c*_ values, 0.01, 0.1, and 0.5, yield 347, 3183, and 15,040 CIPs, respectively.

Although the statistical formula (7) for protein identification is better found than the one (5) we used for microbe identification/classification, we did not switch to the former in this MiCId update for the following reason. When performing microbial identification/classification, one needs to combine hundreds to thousands of peptide database *P* values as opposed to tens of database *P* values for protein identification. For the former case, if one were to use formula (7), the summation becomes very time-consuming to compute and can considerably slow down the entire workflow.

### Microbial Identification

#### Validation of MiCId’s Taxa Identification Using Mixtures of Known Compositions

One important component of our method, as described in the “Statistical Method for Microbial Identification” subsection, is the peptide-driven clustering procedure used to group microbes at different taxonomic levels. The parameters needed for the clustering procedure include the resemblance coefficient, the minimum number of unique CIPs required to prevent a taxon (or a cluster of taxa) from being further clustered, and the condition for taxa to be selected for the next level identification. These parameter values were learned from using BMD-A (as a training dataset) to query DB-1; they are selected by maximizing the “true positive rate” (TPR) at the species level under a specified *E* value cutoff. Once determined, the parameters are used for all level of taxa identifications. When computing the TPR, a cluster of identified microbes contributes only one count; the cluster is viewed as a true positive only if the cluster head is a true positive and as a false positive otherwise. The first half of Table 2 displays, from phylum-level to species-level identifications, the TPR along with the PFD using the parameters learned (with *E* value cutoff 0.01). More pertinent information at the genus and species levels can be found in Supplementary Figure S1.

**Figure 3:**
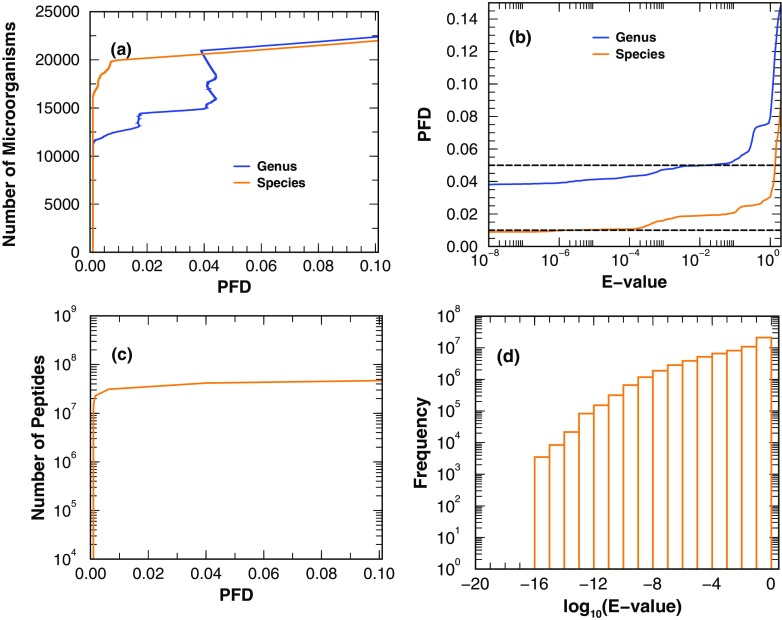
Retrieval assessments using the blended MS/MS dataset BMD-B of known microbe compositions to query DB-1 are shown in panels (**a**) (taxa) and (**c**) (peptides). (**b**) The PFDs versus the *E* values of identified taxa. This panel indicates that using a *E* value cutoff of 0.01, one can control the PFDs at the genus and species level at 5 and 2%, respectively. (**d**) The histogram of peptides identified with different *E* values. BMD-B contains 1400 blended DFs, each of which contains spectra from all 15 genera/species; hence, the maximum count of identifiable genera/species is 21,000. An identified cluster is counted as a true positive only if its cluster head is among the known microbes and a false positive otherwise. The table headings are explained below: SK represents the species key; E[R] is the taxon’s average rank in the identified cluster containing it; IF_1_ is the overall identification fraction (proportion of times a taxon is identified, be it a cluster head or not, from samples containing it); IF_2_ records the identification fraction of a known microbe that also happens to be the head of the cluster it belongs to; IF_3_ reports the identification fraction of a known microbe that not only is the head of the cluster it belongs to but also has unique (taxon-specific) peptide hits; e[NIP] is the average number of identified peptides; e[CS] represents the average cluster size containing the taxon. In the table above, microbial identification was controlled at the 5% PFD. The species keys are 1, *Escherichia coli*; 2, *Enterobacter lignolyticus*; 3, *Streptococcus pyogenes*; 4, *Mycobacterium tuberculosis*; 5, *Salmonella enterica*; 6, *Yersinia pestis*; 7, *Shewanella oneidensis*; 8, *Pseudomonas aeruginosa*; 9, *Bacillus subtilis*; 10, *Bordetella pertussis*; 11, *Bartonella henselae*; 12, *Rhodobacter sphaeroides*; 13, *Thermotoga maritima*; 14, *Geobacter bemidjiensis*; 15, *Caulobacter vibrioides*

With the parameters determined, we use BMD-B and mixture sample datasets of known composition to query DB-1 in order to test the ability of MiCId in microbial identification/classification. The resulting TPR and PFD (with *E* value cutoff 0.01) are displayed in the second half of Table 2. The retrieval curves of taxa and peptides from using BMD-B as queries are plotted in panels a and c of Figure 3. Plotted in panel b of Figure 3 is the PFDs versus the *E* values of identified taxa. This panel indicates that using a *E* value cutoff of 0.01, one can control the PFDs at the genus and species level at 5 and 2%, respectively. Panel d of Figure 3 shows the histogram of peptides identified with different *E* values. BMD-B contains 1400 blended DFs, each of which contains spectra from all 15 genera/species; hence, the maximum count of identifiable genera/species is 21,000. In this assessment, an identified cluster is counted as a true positive only if its cluster head is among the known microbes and a false positive otherwise. Although the table headings are explained in the caption, we shall elaborate on few of them here: IF_1_ is the overall identification fraction (proportion of times a taxon is identified, be it a cluster head or not, from samples containing it); IF_2_ is no larger than IF_1_ as it records the identification fraction of a known microbe that is also the head of the cluster it belongs to; IF_3_ cannot be larger than IF_2_ by definition since it reports the identification fraction of a known microbe that, in addition to satisfying the conditions for IF_2_, must have taxon-specific (unique) peptide hits. The fact that sometimes IF_2_ is smaller than IF_1_ indicates the room for improvement in our identification method: it shows occurrences of a true positive microbe not being the cluster head. There are two scenarios that this can happen. First, it is possible that a false positive microbe having a similar peptidome to the true positive somehow becomes more significant than the true positive microbe and the new cluster head. Second, we can have two true positive microbes sharing a larger number of CIPs and are clustered together.

One quantity of particular interest is IF_2_-IF_3_. Note that with IF_2_-IF_3_ being zero for all genus-level identifications, we know that each microbe present in these DFs has genus-specific peptides identified, indicating separability (or weak correlation) among genera. On the other hand, at the species level, we found several cases with IF_2_-IF_3_ being nonzero (highlighted in orange), indicating that strong similarities exist among certain species. Interestingly, the fact that IF_2_>IF_3_ for several species reveals that MiCId can correctly identify at times these species without relying on species-specific peptides. Another noteworthy point is that even though DB-1 is a fairly large database, the majority of identified peptides are still quite significant (see panel d).

As another test of MiCId’s ability to correctly identify microbes, a series of 85 samples, DFs 39–123, composed of one, two, four, or nine known microbes were used to query DB-1. Panels A and B of Supplementary Figure S2 display the PFD curves for the genus and the species-level identifications, respectively, from samples containing one microbe and several microbes. Panels C and D show the histograms of peptides identified with different *E* values, respectively, from samples containing one microbe and several microbes. The identification performance of MiCId using DFs 39–123 and with the PFD cutoff at 5% is summarized in the associated table of Supplementary Figure S2.

We have also tested if MiCId can identify viruses from samples of Calu-3 human lung cancer cells (infected with influenza A, harvested 0, 3, 7, 12, 18, and 24 h post infection with five replicates at each time point). These samples, DFs 137–226, are from cell lysates using multidimensional protein identification technology (MudPIT) [71] and were not enriched for virus proteins. Using these samples, MiCId was able to correctly identify influenza A as early as 7 h for 2 out of 15 samples and could correctly identify influenza A after 12 h for all of them. Note that with the *E* value cutoff of 0.01, MiCId does not report any false positive regardless whether true positives are reported or not, indicating a robust false positive control. The results obtained is quite surprising given that the size of DB-1 (covering 46,838,064 proteins) is so much larger than that of influenza A (11 proteins), see Supplementary Table S6.

#### Microbial Identification in Complex Mixture of Unknown Composition

**Figure 4:**
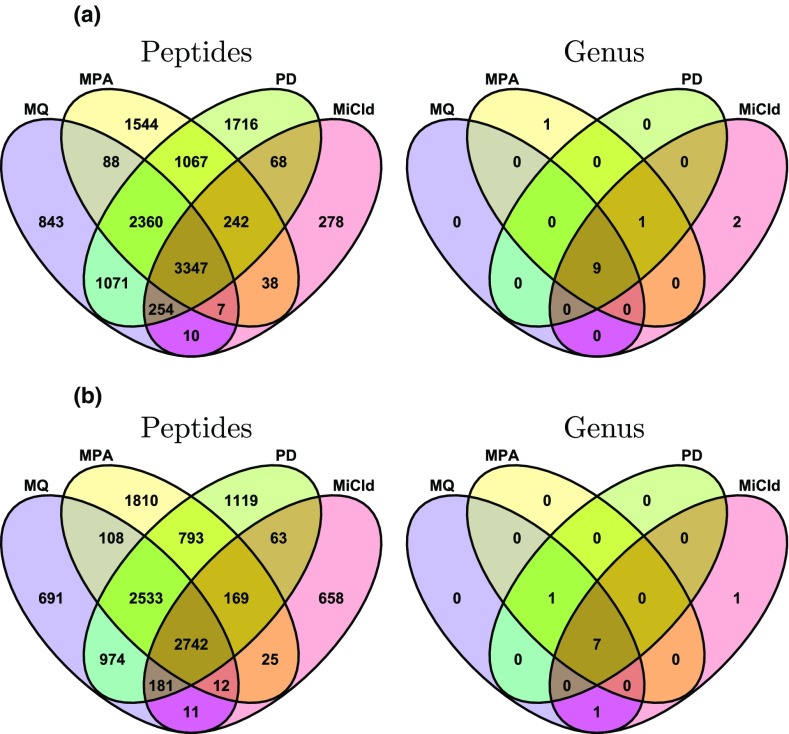
Identification result comparison between MiCId and different workflows by analyzing DF 124 (human stool sample) in two databases DB-3 (**a**) and DB-4 (**b**). The workflows compared with MiCId include MetaProteome analyzer (MPA) that uses X!Tandem for peptide identification [55], MaxQuant (MQ) [55], and proteome discoverer (PD) which uses Sequest-HT and percolator for peptide identification [55]. Plotted in the Venn diagrams are the intersection of nonredundant peptides identified at the 1% PFD for the four workflows. For MQ, MPA, and PD, genus identifications were done by sending all peptides identified at 1% PFD to Unipept with the filtering strategy recommended by [51] enforced. For MiCId, all peptides identified with *E* value ≤ 1 are used for genus identifications, and only heads of genus clusters identified with *E* value ≤ 0.01 are used to generate the Venn diagram

Using a human stool sample, DF-124, we compare the microbial identifications at the genus level of MiCId with the results from a previous study [55] that includes three workflows: MetaProteome Analyzer (MPA) that uses X!Tandem for peptide identification [55], MaxQuant (MQ) [55], and Proteome Discoverer (PD) which uses Sequest-HT and Percolator for peptide identification [55]. In Figure 4, two databases, DB-3 and DB-4, are employed for identification comparison, shown as Venn diagrams, among the four workflows. We did not compare MiCId with the three aforementioned workflows (MQ, MPA, and PD) adapting MEGAN [50] since the authors of [51] have shown that Unipept outperforms MEGAN in terms of pathogen identifications. For MQ, MPA, and PD, genus identifications were done by sending all peptides identified at 1% PFD to Unipept with the filtering strategy recommended by [51] enforced: when the number of unique peptides mapped to a taxon is less than 0.5% of the total number of unique (taxon-specific) peptides, that taxon is viewed as a false positive. For MiCId, all peptides identified with *E* value ≤ 1 are used for genus identifications, and only heads of genus clusters identified with *E* value ≤ 0.01 are used to generate the Venn diagram. Species-level identification comparison was not possible because Unipept processes the database differently at this level [49]. However, more comparisons with the PD workflow adapting Unipept are available in Supplementary Table S7 and Supplementary Figure S3. From Supplementary Figure S3, the readers will notice that although PD in general identifies more peptides than MiCId in this complex sample, MiCId nevertheless identifies more genera. Given the rigorous statistical foundation of MiCId and the low *E* value cutoff used, the additional genera identified by MiCId are unlikely to be false positives. On the other hand, the large number of peptides identified by PD, but not by MiCId, do not yield additional confident genera identifications. This might be because these peptides are primarily false hits or the aforementioned filtering strategy, due to its heuristic nature, sometimes can be too aggressive [47, 51] and lose true positives.

**Figure 5:**
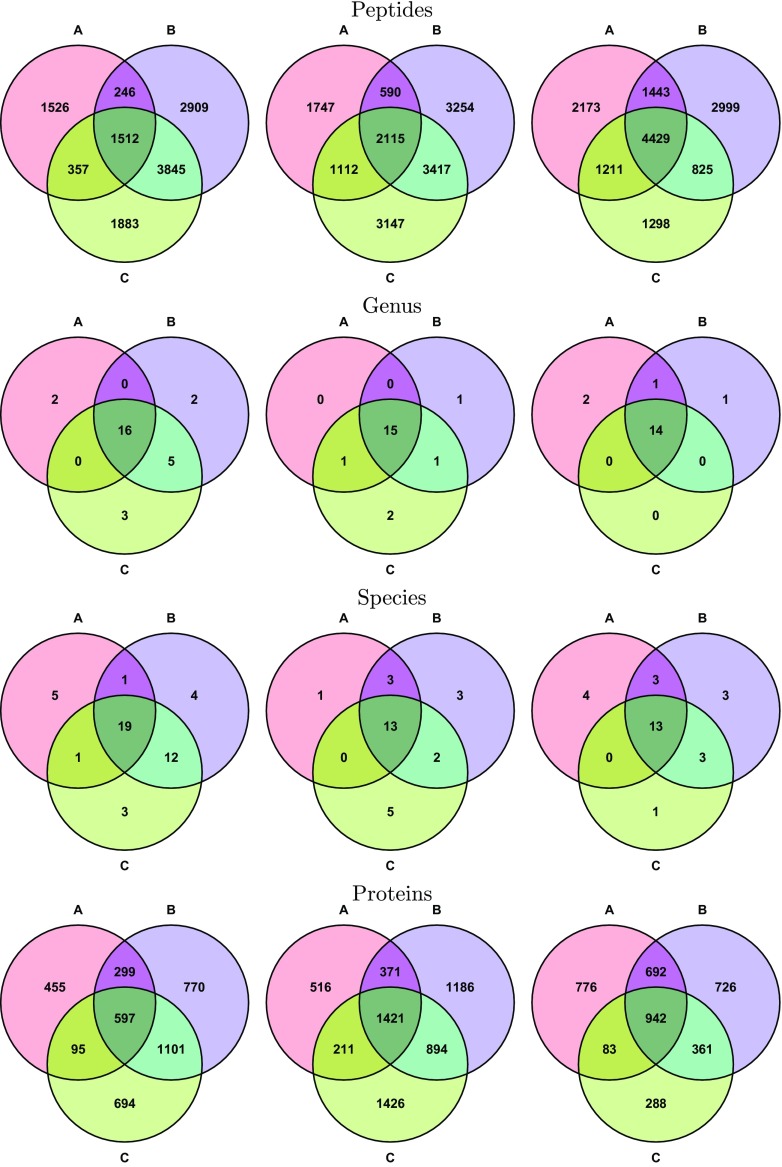
Identification overlaps of peptides, microbes, and proteins among technical replicates. Analyzing nine MS/MS datasets, DFs 128–136, three technical replicates from human stool samples of three different volunteers [30], we plot in the Venn diagrams the overlaps among triplicates in terms of peptides, genera, species, and proteins identified by MiCId when searching DB-2. The peptide Venn diagrams show the intersections of nonredundant peptides identified at the 1% PFD, while other Venn diagrams are constructed using genera, species, and proteins identified with *E* value ≤ 0.01

Note that in Figure 4, although the peptides identified do not have strong overlaps, the genera identified seem to overlap much more. The discrepancy in peptides identified might be attributed to the difference in terms of how PFDs are estimated. Leaving this issue aside, however, a more fundamental question remains: using a workflow such as MiCId (that yields accurate PFDs) to analyze data from several replicates of the same sample, how well can the identified taxa overlap given the possible intrinsic variation due to data-dependent acquisition of typical MS/MS practice? To investigate the size of this intrinsic variation and its impact on taxa identifications, we analyze nine MS/MS datasets DF-128 to DF-136, three technical replicates from human stool samples of three different volunteers [30]. In Figure 5, we plot in the Venn diagrams the overlaps among triplicates in terms of peptides, genera, species, and proteins identified by MiCId when searching DB-2. The peptide Venn diagrams, displaying the intersections of nonredundant peptides identified at the 1% PFD, show a lot more variations than the Venn diagrams for genera and species. This indicates that the taxa identified remain largely the same even though there exists nonnegligible intrinsic variations among technical replicates in terms of peptides/proteins identified [30].

The other problem of interest is to compare the taxa identification performance of various workflows given the same list of input peptides. Evidently, when the input peptides are the same, then except for MiCId, the taxa identified by MQ, MPA, and PD become identical (as they all are given by Unipept). In this context, it becomes the taxon identification comparison between MiCId and Unipept. To meaningfully compare MiCId with Unipept (with and without the filtering strategy proposed in [51]), however, the true positive microbes must be known. We thus used a data set (DF-100 to DF-108) from a mixture of four known microbes. The results are summarized in Table 1. We found that MiCId consistently identify more true positives while controlling the false positives at a lower rate when compared to Unipept. One important observation is that the *heuristic* filtering strategy [51] does not always control the number of false positives to a low number while our statistical method does. It is possible that the *E* value cutoff of 1 in Table 1 may include too many peptides with poor statistical significance, hence hindering the performance of Unipept. For this reason, we have provided in the Supplementary Table S8 the results from controlling the input peptides at 1% PFD using the MiCId statistics. As one may see in the Supplementary Table S8, the same trend persists.

**Table 1:** Genus Identification Comparison Between MiCId and Unipept

Genus assignment for data files 100–108									
		MiCId				Unipept			
DF	NPU	TP_*f*_	TP_*u*_	FP_*f*_	FP_*u*_	TP_*f*_	TP_*u*_	FP_*f*_	FP_*u*_
100	9229	4	4	0	2	3	4	18	591
101	9865	4	4	0	0	3	4	18	634
102	9666	4	4	0	0	3	4	12	595
103	23,248	4	4	0	3	3	4	11	862
104	22,496	4	4	0	1	3	4	11	858
105	23,105	4	4	0	0	3	4	13	870
106	21,730	4	4	0	0	3	4	12	880
107	24,091	4	4	0	1	3	4	13	892
108	21,093	4	4	0	1	3	4	16	866

DF-100 to DF-108, sample mixtures composed of four bacteria *S. pneumoniae*, *S. aureus*, *E. coli*, and *P. aeruginosa*, are used to query DB-1. All peptides with *E* values ≤ 1 are used as input for both MiCId and Unipept. The data file (DF) index is shown in the first column. The number of peptides used (NPU) is displayed in the second column. For Unipept results, the subscript *u* (or *f*) of the number of true positive (TP) and the number of false positive (FP) means that the filtering strategy [51] is turned off (or on); for MiCId, the subscript *u* includes taxa with *E* value ≤ 1 while *f* includes only taxa with *E* value ≤ 0.01

#### Sensitivity Does Not Always Decrease as the Database Size Increases

Sensitivity drop due to searching a large database has been examined and reported by many groups [30, 32, 35]. The basic fact is that when a simple (single) microbe sample is used, searching a smaller database (containing the proteome of that microbe) has a better sensitivity than searching a larger database [30]. However, when a more complex sample is used, searching a larger database has the advantage of discovering unexpected but perhaps true positive peptides [35, 55]. In other words, even though searching a larger database reduces the statistical significances of peptides previously identified in a smaller database, new peptides not contained in the smaller database may be identified with high significance. Hence, the sensitivity in terms of peptide identification may even increase when searching a larger database.

**Figure 6:**
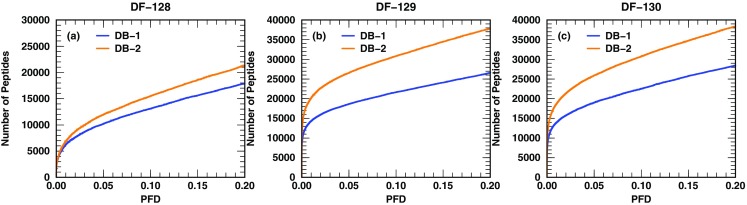
Peptide retrieval curves for DFs 128–130 (complex samples) when searching DB-1 (46 GB) and DB-2 (200 GB). At the same PFD value, more peptides are identified when searching the larger database (DB-2)

To investigate the latter point, we have used data from complex samples (DFs 128–136) to query DB-1 and DB-2. The sizes of DB-1 and DB-2 are 46 and 200 GB, respectively. The 46,838,064 protein sequences (807,574,956 nonredundant tryptic peptides) in DB-1 are from 23,911 organisms, belonging to 13,072 species, 1890 genera, and 517 families. The 260,931,852 protein sequences (2,507,889,685 nonredundant tryptic peptides) in DB-2 are from 75,356 organisms, belonging to 26,368 species, 2870 genera, and 701 families. In terms of number of tryptic peptides, DB-2 is about three times the size of DB-1, although in terms of protein sequences it is about 5.6 times the size of DB-1. The peptide retrieval curves for each DFs are shown in groups. The first group, containing PFD curves from DFs 128–130, is displayed in Figure 6. The other two groups are shown in Supplementary Figure S4. The consistent trend for each of these DFs (from complex samples) is that at a given PFD value, searching the larger database (DB-2) indeed yields a larger number of identified peptides in comparison with searching the smaller database (DB-1).

### Protein Identification

MiCId performs protein identifications by extending its taxa identifications in one go. It first queries the MS/MS spectra in a peptide-centric database, which is constructed from microbial protein sequences and used for microbial identification at various taxonomic levels. MiCId then uses the protein sequences belonging to the confidently identified microbial species to construct a protein database on-the-fly and queries in it the MS/MS spectra again for protein identification. This approach differs from metaproteomic approaches which identify proteins directly from identified peptides without performing microbial identifications first [30, 48]. Because each on-the-fly constructed protein database, no longer covering all microbes, is much smaller than the original peptide-centric database, MiCId can consider post-translationally modified peptides and semi-enzymatic (semi-tryptic) peptides during protein identification without adding much computational cost. In a previous study [65], we have shown that the method employed for protein identification in MiCId assigns accurate statistical significance to identified proteins and also has a protein retrieval performance that is no worse than that of any other protein identification method. Therefore, we only present the protein identification results of MiCId from analyzing DFs 128–136, three technical replicates from human stool samples of three different volunteers [30]). In the last row of Figure 5, we plot in the Venn diagrams the overlaps among triplicates in terms of proteins identified by MiCId when searching DB-2. As one may see, there exists nonnegligible variation among the triplicate results in terms of proteins identified. This may be attributed to the significant variations in the peptides identified, see the Venn diagrams on the first row of Figure 5. On the other hand, from the Venn diagrams on the second row (genus) and the third row (species) of Figure 5, the genus and species identifications appear consistent despite the variations in peptides/proteins identified.

When clustering proteins, MiCId does not use protein homology to cluster them. Rather, it uses a peptide-centric clustering procedure which is described below: first peptides identified with *E* values ≤ 1 are mapped to proteins in the database; second, a standardized weighted count (*Z*) is assigned to each identified peptide (whose *E* value is *E*), $Z(E)=1/\left(1+\frac{E}{E_c}\right)$, where *E*_*c*_ is the *E* value cutoff used to control peptide identification at a 5% PFD value; third, the sum, *W*_*p*_, of the weighted (*Z*) peptide evidence for every protein *p* is computed and used to sort proteins in order of decreasing *W*; fourth, one starts with the worst ranking protein *p* and clusters it to the better ranking protein which can explain the highest percentage of *W*_*p*_. If that percentage is below 95%, *p* does not cluster into any better ranking protein; fifth, continue the process in step 4 for the second worst ranking protein and repeats it until one has tested the second best ranking protein. However, there is an exception: a protein will not cluster if it has a unique evidence peptide (a peptide that maps to only one protein) with assigned *E* value less than both 10^−4^ and 1/*n*_*s*_, with *n*_*s*_ being the number of spectra collected per experiment. Specifically, we cluster proteins that share the majority of the peptide evidences when the information obtained from identified peptides does not allow us to pin point a specific protein. Because we do not assign functionality to proteins identified, we do not cluster proteins by their homology. In fact, it is possible for two totally dissimilar proteins to be clustered together if they share a couple of peptides that happened to be identified with high confidence.

### Execution Time

**Figure 7:**
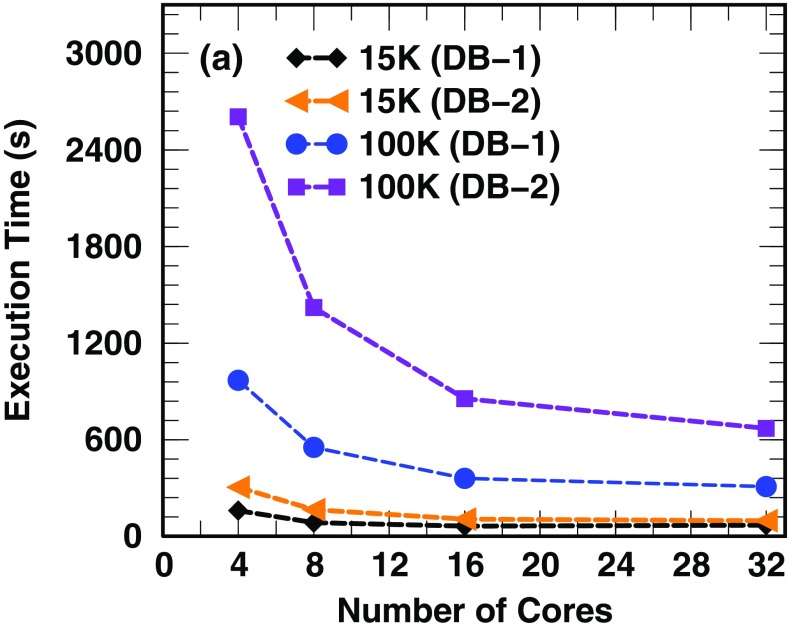
MiCId execution time versus number of cores using two datasets (one containing 15,000 MS/MS spectra and the other 100,000 MS/MS spectra) to query DB-1 (46 GB) and DB-2 (200 GB)

With speed a main consideration, MiCId code was written in C++ and its routine for database search was written using parallel programming. This allows users to run jobs with a flexible number of cores. We have measured the execution time of MiCId in performing microbe identification when querying MS/MS spectra in DB-1 (23,911 organisms and 46 GB) and DB-2 (75,356 organisms and 200 GB). Figure 7 shows that for data sets of ≈100,000 MS/MS spectra, microbe identification using DB-1 can be accomplished in about 15 min with 4 cores and reduces to around 6 min with 16 cores. On the other hand, with ≈100,000 MS/MS spectra, the execution time for microbe identification using DB-2 ranges from around 44 min (with 4 cores) to 14.2 min (with 16 cores). Our results indicate that when the database size increases by a factor of 4.3, the execution time increases only by a factor of 2.4 (using 16 cores), suggesting that only a 5.6-fold execution time will be encountered with a 10-fold database size increase. This reflects the scalability of MiCId in handling large databases. Figure 7 also shows that the execution time reduction by increasing the number of cores appears to reach a plateau at 16 cores. This is because the piece of C++ code performing microbial identification has not yet been parallelized, thus incurring a constant time cost.

## Limitation and Future Direction

We have mentioned that based on Figure 1, it appears that unambiguous identification at the species level for certain species can be challenging. This difficulty worsens when complex samples are concerned and presents the direction for future efforts. For example, most real environmental samples will not be clonal, but might be clades of related organisms. Hence, even the concept of “species” can be problematic here. Separating closely related organisms evolved from a common ancestor species may not be possible under our current method due to lack of proteome information and high degree of proteome similarities of closely related organisms. Nevertheless, identifying the ancestral species can be a realistic long-term goal.

On the other hand, numerous lines of evidence indicate that individual “strains” might differ in physiology, and thus, detection at strain levels might be needed for clinical applications. For this case, however, cultured colonies of bacteria might be possible and progress has been made at the strain-level identifications in simple mixtures [45]. The challenge and future direction here pertains to how to minimize the culture time and increase the identification rate.

Evidently, limitation of our method exists. In fact, interpretation of the results using our analysis workflow depends on the presence/absence of the correct microorganism in the database. If one is certain that the correct microorganism is present in the database, one should interpret the results as microbial identification. On the other hand, if one is sure that the correct microorganism is absent from the database, one may interpret the results as microbial classification, i.e., finding the closest relative.

## Conclusion

In this study, we have proposed a workflow for microbial classification/identification by processing MS/MS data acquired in high-resolution mass spectrometers. We have used a large number of MS/MS data files to assess MiCId’s ability in identifying microbes in mixture samples. Results from our identification assessment show that (at the *E* value cutoff 0.01 yielding PFD ≤ 5%) MiCId has an average true positive rate of 0.9813 at the genus level and 0.9550 at the species level. (More details can be found in Table 2.) One should note, however, that these numbers were obtained from blended spectra of single species samples. Generalization to complex microbiota samples must be taken with a grain of salt. Nevertheless, we were also able to show, by comparing with published results, that MiCId performs comparably to existing methods in microbial identifications. A major advantage of MiCId over existing methods is that it can assign to microbes and proteins identified accurate statistical significance, e.g., *E* values, thus providing users a measure suitable for controlling false positives (type I errors) and to estimate the PFD via the Sorić equation [72]. In contrast with current metaproteomics methods, MiCId’s protein identification strategy begins with microbial identifications, allowing users to consider post-translationally modified peptides and semi-enzymatic (semi-tryptic) peptides without adding much computational cost. This feature might be welcomed by researchers interested in proteomics of complex microbial mixtures. Storing only nonredundant peptides from proteins digested in silico, the peptide-centric database of MiCId has a database size to number of organism ratio that decreases with the increasing number of organisms, making database search time increase only sublinearly with the number of the included organisms. MiCId’s workflow is fully automated: users need only specify a list of microbes to be included in the peptide-centric database and the parameters to analyze the MS/MS spectra, and everything is handled internally by MiCId. MiCId can be downloaded freely at http://www.ncbi.nlm.nih.gov/CBBresearch/Yu/downloads.html.

**Table 2:** True Positive Rate (TPR) and Proportion of False Discoveries (PFD) at the 0.01 *E* Value Cutoff Using BMD-A and BMD-B

True positive rate and proportion of false discoveries						
MS/MS data set BMD-A						
Taxonomical level	P	C	O	F	G	S
TPR_*Ec* = 0.01_	100.00%	94.13%	99.99%	99.93%	97.69%	94.10%
PFD_*Ec* = 0.01_	3.02%	3.47%	2.33%	2.59%	1.49%	1.91%
MS/MS data set BMD-B						
Taxonomical level	P	C	O	F	G	S
TPR_*Ec* = 0.01_	100.00%	100.00%	100.00%	100%	98.57%	96.90%
PFD_*Ec* = 0.01_	0.07%	0.00%	0.15%	2.37%	4.98%	1.89%

The first half of the table displays the TPR along with the PFD (with *E* value cutoff 0.01), using the parameters learned from BMD-A and apply them to BMD-A, at various taxonomic identification levels: phylum (P), class (C), order (O), family (F), genus (G), and species (S). Displayed in the second half of the table are the microbial identification/classification results using BMD-B to query DB-1 with the parameters learned from using BMD-A

The study presented here focuses mainly on the classification/identification with accurate statistical significance for microbial mixtures via high-resolution MS/MS spectra. True advances in microbial studies using complex samples, however, require multidisciplinary collaboration. Along this direction, many innovative approaches have been developed. Such examples include but are not limited to faster MS/MS instruments like Orbitrap Fusion Lumos Tribid [73–75] that can better sample complex mixtures by collecting a greater number of MS/MS spectra, improved sample preparation protocols for pathogenic samples [76, 77], and new techniques to isolate strain-specific peptides from microbes’ membrane proteins [78, 79]. These advances, together with computational workflows (such as MiCId) built on firm statistical foundations, may potentially further improve microbe identification in complex samples.

## Electronic Supplementary Material

ESM 1 (PDF 262 kb)

ESM 2 (XLSX 2181 kb)

ESM 3 (XLSX 2195 kb)

ESM 4 (CLS 46 kb)
