# Supplementary material for: Rapid Classification and Identification of Multiple Microorganisms with Accurate Statistical Significance via High-Resolution Tandem Mass Spectrometry
Source: J Am Soc Mass Spectrom. 2018 Jun 5;29(8):1721–37. doi: 10.1007/s13361-018-1986-y (PMC6061032; doi:10.1007/s13361-018-1986-y)
Supplement: Supplementary file 1 — (PDF 262 kb) [file 13361_2018_1986_MOESM1_ESM.pdf]

Noname manuscript No.
(will be inserted by the editor)

Electronic Supplementary Material for "Rapid Classification and Identification with Accurate Statistical Significance for Microorganisms in Mixtures via High Resolution Tandem Mass Spectrometry"

Gelio Alves[1] · Guanghui Wang[2] · Aleksey Y. Ogurtsov[1] · Steven K. Drake[3] · Marjan Gucek[2] · David B. Sacks[4] · Yi-Kuo Yu[1⋆]

[1]**National Center for Biotechnology Information, National Library of Medicine, National Institutes of Health, Bethesda, MD 20894, USA**
[2]**Proteomics Core, National Heart, Lung, and Blood Institute, National Institutes of Health, Bethesda, MD 20892, USA**
[3]**Critical Care Medicine Department, Clinical Center, National Institutes of Health, Bethesda, MD 20892, USA**
[4]**Department of Laboratory Medicine, Clinical Center, National Institutes of Health, Bethesda, MD 20892, USA**

Received: date / Accepted: date

Supplementary Figures

⋆ *Correspondence to:* Yi-Kuo Yu; *email:*yyu@ncbi.nlm.nih.gov

National Center for Biotechnology Information, National Library of Medicine, NIH, Bethesda, MD 20894, USA

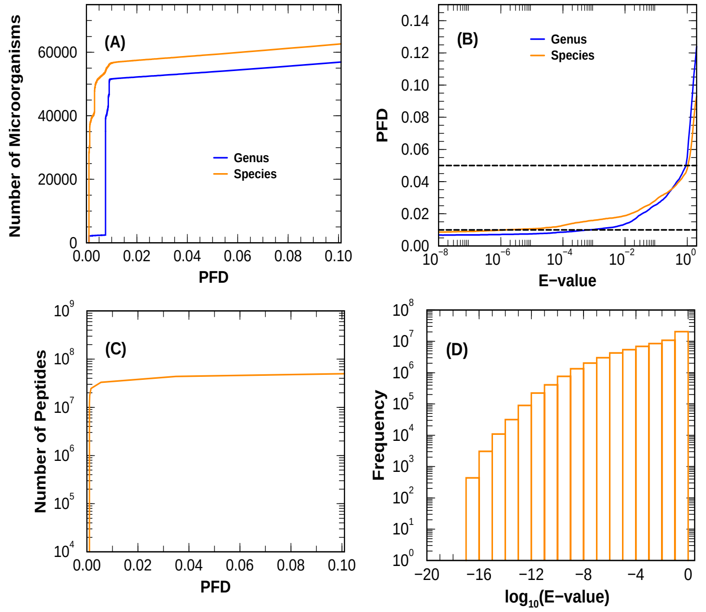

Species level							Genus level						
SK	E[R]	IF_1	IF_2	IF_2-IF_3	E[NIP]	E[CS]	SK	E[R]	IF_1	IF_2	IF_2-IF_3	E[NIP]	E[CS]
1	1.0000	100.00	100.00	0.00	605.25	1.01	1	1.0000	100.00	100.00	0.00	949.48	1.06
2	1.0000	100.00	100.00	0.00	419.22	1.00	3	1.0012	100.00	99.96	0.00	906.53	1.43
3	1.3176	100.00	68.24	4.89	639.72	3.20	5	1.0000	100.00	100.00	0.00	671.17	1.05
4	1.5439	100.00	45.65	7.81	385.28	3.33	7	1.1296	99.80	92.95	0.00	827.86	2.4
5	1.0278	96.40	93.71	0.00	468.46	1.03	8	1.2805	99.80	74.97	15.42	796.67	3.90
6	1.0020	100.00	99.84	0.00	546.61	1.06	9	1.0000	100.00	100.00	0.00	397.63	1.00
7	1.2755	99.72	93.67	0.92	815.98	2.19	10	1.0000	100.00	100.00	0.00	457.59	1.00
8	1.1457	99.76	91.55	32.68	838.99	8.65	11	1.0008	100.16	100.08	0.20	680.10	1.86
9	1.3100	100.00	85.50	25.47	330.12	7.00	12	1.0000	100.00	100.00	0.00	599.61	1.00
10	1.0000	100.00	100.00	0.92	376.72	1.08	13	1.0000	100.00	100.00	0.00	411.19	1.00
11	1.0000	100.00	100.00	0.04	674.31	1.07	14	1.0000	100.00	100.00	0.00	458.73	1.00
12	1.0036	100.00	99.68	80.94	484.04	4.73	15	1.1571	88.99	84.98	0.00	771.86	1.34
13	1.0000	100.00	100.00	0.00	377.85	1.00	16	1.0000	100.00	100.00	0.00	283.10	1.00
14	1.0000	100.00	100.00	0.00	418.63	1.00	17	1.0000	100.00	100.00	0.00	474.75	1.00
15	1.1010	86.42	85.26	0.00	715.04	1.58	18	1.0000	100.00	100.00	0.00	224.19	1.01
16	1.0008	100.00	99.92	1.44	251.10	1.05	19	1.0000	100.00	100.00	0.00	390.48	1.00
17	1.0000	100.00	100.00	0.00	474.75	1.00	20	1.0000	100.00	100.00	0.08	191.08	1.00
18	1.0000	100.00	100.00	0.00	207.09	1.00	21	1.0000	100.00	100.00	0.00	194.50	1.00
19	1.0000	100.00	100.00	0.00	364.90	1.00	22	1.0000	100.00	100.00	0.00	300.30	1.00
20	1.0252	99.96	97.92	20.66	175.68	2.47	23	1.0000	100.00	100.00	0.08	386.45	1.00
21	1.0000	100.00	100.00	0.00	191.31	1.00	24	1.0000	98.84	98.84	1.00	170.21	1.00
22	1.0000	100.00	100.00	0.08	283.10	1.00							
23	1.0000	99.92	99.92	0.16	278.82	1.00							
24	1.0000	98.08	98.08	3.36	125.12	1.01							

Fig. S1: Assessment of the clustering parameters learned using the blended MS/MS dataset BMD-A to query DB-1 are shown in panels A (taxa) and C (peptides). Panel B displays the PFDs versus the *E*-values of identified taxa. This panel indicates that using a *E*-value cutoff of 0.01, one can control the PFDs at the genus and species level at 2% and 1% respectively. Panel D shows the histogram of peptides identified with different *E*-values. BMD-A contains 2500 blended MS/MS DFs, each of which contains spectra from all 24 species (21 genera); hence the maximum total count is 60000 (52500) at the species (genus) level. Only microbes identified at PFD $\leq$ 5% are included in this table. The table headings are explained below: SK represents the species key; E[R] is the taxon's average rank in the identified cluster containing it; IF_1 is the overall identification fraction (proportion of times a taxon is identified, be it a cluster head or not, from samples containing it); IF_2 records the identification fraction of a known microbe that also happens to be the head of the cluster it belongs to; IF_3 reports the identification fraction of a known microbe that not only is the head of the cluster it belongs to but also has unique peptide hits; E[NIP] is the average number of identified peptides; E[CS] represents the average cluster size containing the taxon. The species keys are: 1, *Rhodobacter sphaeroides*; 2, *Rhodobacter capsulatus*; 3, *Yersinia pestis*; 4, *Yersinia pseudotuberculosis*; 5, *Sinorhizobium medicae*; 6, *Sinorhizobium meliloti*; 7, *Salmonella enterica*; 8, *Escherichia coli*; 9, *Mycobacterium tuberculosis*; 10, *Shewanella oneidensis*; 11, *Anabaena variabilis*; 12, *Bacillus subtilis*; 13, *Ruminiclostridium thermocellum*; 14, *Desulfovibrio alaskensis*; 15, *Enterobacter lignolyticus*; 16, *Geobacter bemidjiensis*; 17, *Kineococcus radiotolerans*; 18, *Novosphingobium aromaticivorans*; 19, *Candidatus Pelagibacter.ubique*; 20, *Thermotoga maritima*; 21, *Kosmotoga olearia*; 22, *Caulobacter vibrioides*; 23, *Pseudomonas aeruginosa*; 24, *Bartonella henselae.*

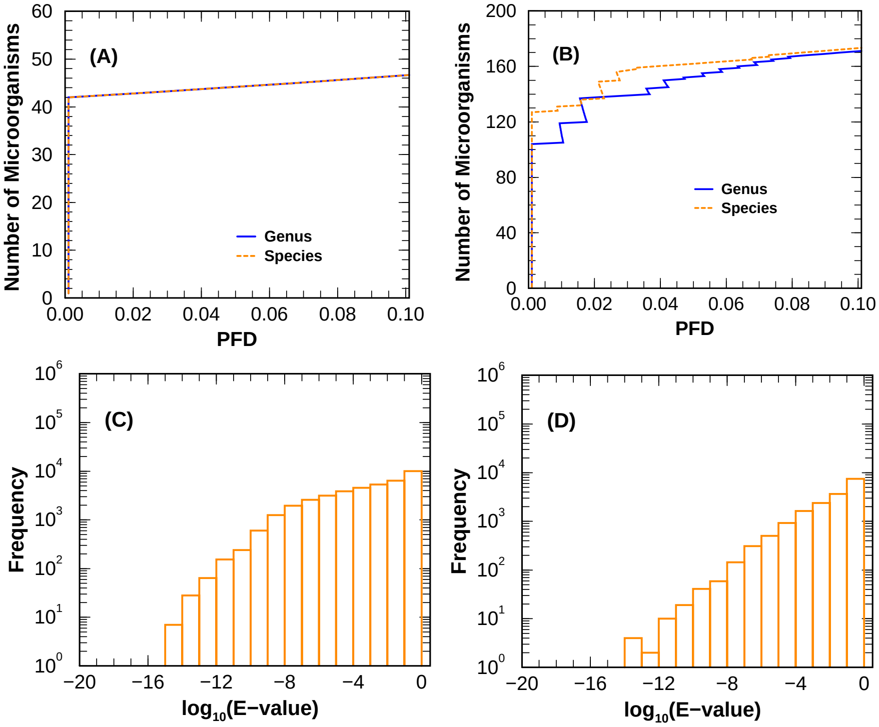

Species (Single Microorganism Samples)							Species (Multi-Microorganism Samples)						
SK	E[R]	IF_1	IF_2	IF_3	E[NIP]	E[CS]	SK	E[R]	IF_1	IF_2	IF_3	E[NIP]	E[CS]
1	1.00	7/7	7/7	7/7	1776.00	5.57	1	1.00	26/27	26/27	19/27	339.15	4.38
2	1.00	3/3	3/3	3/3	1516.33	1.00	2	1.00	9/9	9/9	9/9	632.11	1.00
3	1.00	3/3	3/3	3/3	1365.33	1.00	3	1.00	9/9	9/9	9/9	872.78	1.00
4	1.00	3/3	3/3	3/3	1110.33	1.00	4	1.00	21/21	21/21	21/21	533.86	1.00
5	1.00	3/3	3/3	3/3	1063.00	1.00	5	1.00	26/29	26/29	24/29	440.62	1.62
6	1.00	3/3	3/3	3/3	1110.67	1.00	6	1.00	32/33	32/33	31/32	409.41	1.19
7	1.00	4/4	4/4	4/4	1568.25	7.00	12	1.00	2/6	2/6	2/6	1085.00	2.00
8	1.00	4/4	4/4	4/4	1507.25	1.00	13	1.00	6/6	6/6	6/6	516.50	1.00
9	1.00	4/4	4/4	4/4	1838.25	2.00	14	1.00	4/6	4/6	4/6	161.25	1.00
10	1.00	4/4	4/4	4/4	1634.25	2.75	15	1.00	4/4	4/4	4/4	143.25	1.00
11	1.00	4/4	4/4	4/4	1863.00	1.00	16	1.00	4/4	4/4	4/4	134.50	1.00
							17	1.00	4/4	4/4	4/4	124.75	1.00
							18	1.00	4/4	4/4	4/4	295.25	1.00
							19	1.00	3/4	3/4	3/4	19.33	1.00

Fig. S2: Panels A and B display the PFD curves for the genus and the species level identifications respectively from samples containing one microbe (DFs 39-80) and several microbes (DFs 81-123). Panels C and D show the histograms of peptides identified with different E-values respectively from samples containing one microbe and several microbes. The database used is DB-1. There are 42 DFs from samples containing one microbe, hence the maximum count of identifiable species is 42 (which is the sum of all denominators of the IF on the left half of the table). The other 43 DFs are from mixtures of 2, 4, or 9 microbes and the maximum count of identifiable species is 166 (which is the sum of all denominators of the IF on the right half of the table). The table headings are explained below: SK represents the species key; E[R] is the taxon's average rank in the identified cluster containing it; IF_1 is the overall identification fraction (proportion of times a taxon is identified, be it a cluster head or not, from samples containing it); IF_2 records the identification fraction of a known microbe that also happens to be the head of the cluster it belongs to; IF_3 reports the identification fraction of a known microbe that not only is the head of the cluster it belongs to but also has unique peptide hits; E[NIP] is the average number of identified peptides; E[CS] represents the average cluster size containing the taxon. Only microbes identified with PFD $\leq$ 0.05 were included in the Table. The species keys are: 1, *Escherichia coli*; 2, *Moraxella catarrhalis*; 3, *Haemophilus influenzae*; 4, *Pseudomonas aeruginosa*; 5, *Streptococcus pneumoniae*; 6, *Staphylococcus aureus*; 7, *Mycobacterium tuberculosis*; 8, *Salmonella enterica*; 9, *Yersinia pestis*; 10, *Yersinia pseudotuberculosis*; 11, *Shewanella oneidensis*; 12, *Enterococcus faecalis*; 13, *Pasteurella multocida*; 14, *Lactobacillus acidophilus*; 15, *Brevibacillus laterosporus*; 16, *Lactobacillus casei*; 17, *Pediococcus pentosaceus*; 18, *Saccharomyces.cerevisiae*; 19, *Rhodotorula glutinis*/*Rhodotorula toruloides*.

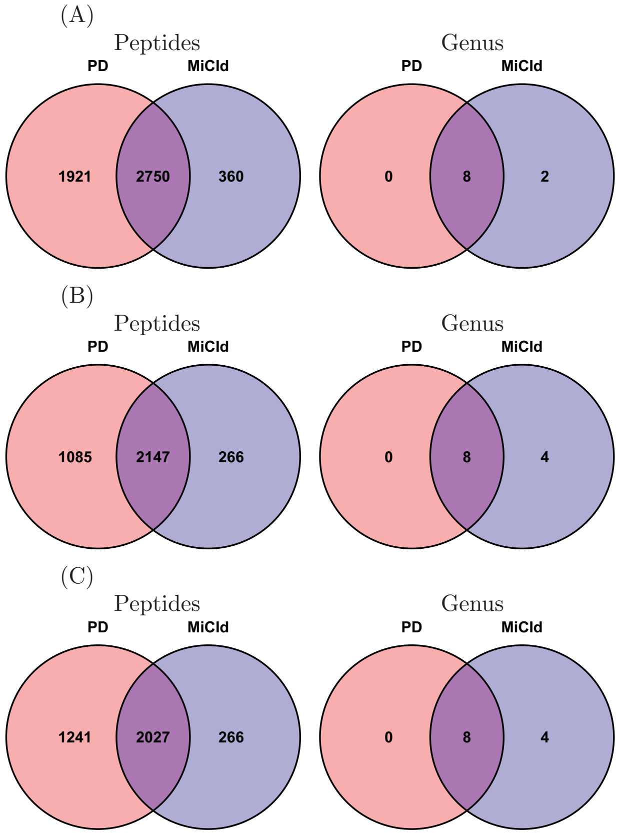

Fig. S3: Identification results overlaps of MiCId and Proteome Discoverer (PD) when querying database DB-5 with DFs 125-127 (human stool samples). Plotted in the Venn diagrams are the intersections of non-redundant peptides identified at the 1% PFD. For PD genus identification was done by sending all peptides identified at 1% PFD to Unipept with the filtering strategy recommended by [1] enforced. For MiCId all peptides identified with E-value ≤ 1 are used for genus identifications, and only heads of genus clusters identified with E-value ≤ 0.01 are used to generate the Venn diagrams.

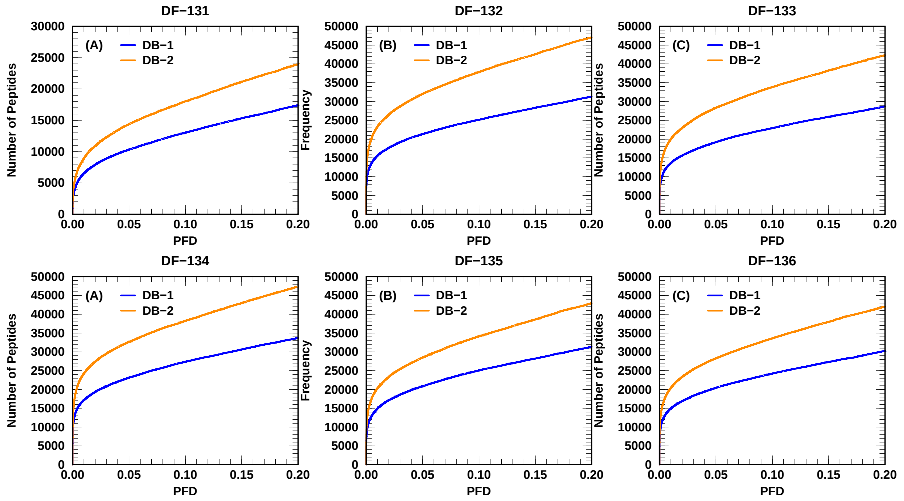

Fig. S4: Peptide retrieval curves for DFs 131-136 (complex samples) when searching DB-1 (46GB) and DB-2 (200GB). At the same PFD value, more peptides are identified when searching the larger database (DB-2).

Supplementary Tables

Table S1: The list of LC-MS/MS data files (DF's) downloaded from the ProteomeXchange at http://www.proteomexchange.org/ and from PeptideAtlas at http://www.peptideatlas.org/.

MS/MS data used for blended MS/MS dataset (BMD-A)			
Sample ID PXD001860			
DF	Data File Name	Species	# MS/MS
1	RSPH_Ponly_16_B_8May12_Earth_12-03-38.mzML	*Rhodobacter sphaeroides*	18876
2	R_cap_cckA_02_Run4_16Nov11_Earth_11-07-28.mzML	*Rhodobacter capsulatus*	15092
3	SBEP_YPCO_023_R1_16Sep10_Falcon_10-07-37.mzXML	*Yersinia pestis*	11915
4	SBEP_YSTB_015_R3_10Oct10_Falcon_10-07-40.mzXML	*Yersinia pseudotuberculosis*	11220
5	Sino_WSM419_G_5_02Apr12_Tiger_12-01-15.mzML	*Sinorhizobium medicae*	20741
6	Sino_1021_G_4_30Mar12_Tiger_12-01-14.mzML	*Sinorhizobium meliloti*	23946
7	SBEP_STM_286_2C_5Apr10_Falcon_10-01-23.mzXML	*Salmonella Typhimurium*	12337
8	Ecoli432_R1-rr_18Dec09_Falcon_09-09-14.mzXML	*Escherichia coli*	12626
9	MtbH37Rv_03_run1_28Mar10_Draco_10-01-17.mzXML	*Mycobacterium tuberculosis*	9470
10	QC_Shew_11-06_2p5_a_28May12_Polaroid_11-12-27.mzXML	*Shewanella oneidensis*	23855
11	Re_Avaria_06_VorbiA_3Sep09_Falcon_09-07-41.mzML	*Anabaena variabilis*	15484
12	WSU_BF_PC_20_R1_15Feb11_Falcon_11-01-01.mzML	*Bacillus subtilis*	9857
13	C.thermocellum_Lysate_CO2a_11Nov11_Earth_11-07-28.mzML	*Ruminiclostridium thermocellum*	14779
14	JW_DSV_022_R1_18Jun10_Andromeda_10-05-09.mzML	*Desulfovibrio alaskensis*	12198
15	E_ligno_SCF1_X_02_Run2_11Nov11_Earth_11-07-26.mzML	*Enterobacter lignolyticus*	15951
16	Gbem_BulkNP_0__block1_7Mar12_Doc_12-02-12.mzML	*Geobacter bemidjiensis*	10260
17	Kradi002_run1_7Aug08_Draco_08-07-15.mzML	*Kineococcus radiotolerans*	11108
18	Mixed_subcell-50c_16Sep10_Falcon_09-11-16.mzML	*Novosphingobium aromaticivorans*	10387
19	P_ubique_SL_09_run2_22Dec10_Falcon_10-11-07.mzML	*Candidatus Pelagibacter ubique*	10613
20	TM_stat_G_R1_R2_20Jan11_Phoenix_10-09-33.mzML	*Thermotoga maritima*	25666
21	K_olearia_50C_Soluble-2_4Apr12_Jaguar_12-02-26.mzML	*Kosmotoga olearia*	10199
22	Caulo_174_2_Caulo_174_20Feb09_Falcon_08-11-04.mzML	*Caulobacter vibrioides*	12868
23	Bhens_Cyt_01_02_Ahrens_14Jul11_Tiger_11-05-30.mzML	*Bartonella henselae*	8436
24	NCRR_PERS_010_24Feb12_Doc_12-02-12.mzML	*Pseudomonas aeruginosa*	14186
MS/MS data used for blended MS/MS dataset (BMD-B)			
Sample ID PXD001860			
24	NCRR_PERS_010_24Feb12_Doc_12-02-12.mzML	*Pseudomonas aeruginosa*	14186
25	MtbH37Rv_03_run2_28Mar10_Draco_10-01-15.mzXML	*Mycobacterium tuberculosis*	9257
26	SBEP_STM_286_2D_15Apr10_Falcon_10-01-24.mzXML	*Salmonella Typhimurium*	12242
27	SBEP_YPCO_022_R3_21Sep10_Falcon_10-07-40.mzXML	*Yersinia pestis*	11260
28	QC_Shew_12_01_2p5_b_15Jun12_Polaroid_11-07-67.mzXML	*Shewanella oneidensis*	66994
28	WSU_BF_PC_20_R2_24Feb11_Falcon_11-01-02.mzML	*Bacillus subtilis*	11694
30	RSPH_Ponly_16_A_12May12_Earth_12-03-13.mzML	*Rhodobacter sphaeroides*	17273
31	TM_stat_G_R1_R2_20Jan11_Phoenix_10-09-33.mzML	*Thermotoga maritima*	26948
32	Gbem_memsol_013_block1_26Jun12_Falcon_12-06-02.mzML	*Geobacter bemidjiensis*	13231
33	120324_OV4_YL_Bp_3hs_1.raw	*Bordetella pertussis*	14770
34	Bhens_Cyt_01_01_Ahrens_14Jul11_Tiger_11-05-29.mzML	*Bartonella henselae*	8394
35	Caulo_174_1_Caulo_174_20Feb09_Falcon_08-11-04.mzML	*Caulobacter vibrioides*	13102
36	Ecoli432_R2_7Dec09_Falcon_09-09-15.mzXML	*Escherichia coli*	11916
37	E_ligno_SCF1_X_03_Run2_11Nov11_Earth_11-07-26.mzML	*Enterobacter lignolyticus*	15364
Sample ID PAe000283			
38	PAe000283_mzXML_201104200930.tar.gz	*Streptococcus pyogenes*	17366

Table S2: The list of LC-MS/MS data file (DF's) downloaded from the ProteomeXchange at http://www.proteomexchange.org/.

DF	Data File Name	Sample ID
	Escherichia coli	
39	Ecoli432_R1-rr_18Dec09_Falcon_09-09-14.mzXML	PXD001860
40	Ecoli432_R2_7Dec09_Falcon_09-09-15.mzXML	PXD001860
41	Ecoli432_R3_7Dec09_Falcon_09-09-16.mzXML	PXD001860
42	Ecoli432_R4_15Dec09_Falcon_09-09-16.mzXML	PXD001860
	Mycobacterium tuberculosis	
43	MtbH37Rv_03_run1_28Mar10_Draco_10-01-17.mzXML	PXD001860
44	MtbH37Rv_03_run2_28Mar10_Draco_10-01-15.mzXML	PXD001860
45	MtbH37Rv_03_run3_28Mar10_Draco_10-01-15.mzXML	PXD001860
46	MtbH37Rv_04_run1_26Mar10_Draco_10-01-17.mzXML	PXD001860
	Salmonella typhimurium	
47	SBEP_STM_286_2C_5Apr10_Falcon_10-01-23.mzXML	PXD001860
48	SBEP_STM_286_2D_15Apr10_Falcon_10-01-24.mzXML	PXD001860
49	SBEP_STM_287_2C_1Apr10_Falcon_10-01-23.mzXML	PXD001860
50	SBEP_STM_287_2D_20Apr10_Falcon_10-01-24.mzXML	PXD001860
	Yersinia pestis	
51	SBEP_YPCO_022_R1_16Sep10_Falcon_10-07-37.mzXML	PXD001860
52	SBEP_YPCO_022_R2_23Sep10_Falcon_10-07-37.mzXML	PXD001860
53	SBEP_YPCO_022_R3_21Sep10_Falcon_10-07-40.mzXML	PXD001860
54	SBEP_YPCO_023_R1_16Sep10_Falcon_10-07-37.mzXML	PXD001860
	Yersinia pseudotuberculosis	
55	SBEP_YSTB_015_R1_28Sep10_Falcon_10-07-37.mzXML	PXD001860
56	SBEP_YSTB_015_R2_28Sep10_Falcon_10-07-39.mzXML	PXD001860
57	SBEP_YSTB_015_R3_10Oct10_Falcon_10-07-40.mzXML	PXD001860
58	SBEP_YSTB_016_R1_24Sep10_Falcon_10-07-37.mzXML	PXD001860
	Shewanella oneidensis	
59	QC_Shew_12_01_Run-07_21Jun12_Roc_12-04-08.mzXML	PXD001860
60	QC_Shew_12_01_Run-07_18Jun12_Roc_12-04-08.mzXML	PXD001860
61	QC_Shew_12_01_pt5_d_29Jun12_Jaguar_12-02-27.mzXML	PXD001860
62	QC_Shew_12_01_pt5_c_29Jun12_Jaguar_12-02-26.mzXML	PXD001860
	Streptococcus pneumoniae	
63	QE_150508_32.raw	PXD004321
64	QE_150508_35.raw	PXD004321
65	QE_150508_38.raw	PXD004321
	Staphylococcus aureus	
66	QE_150611_128.raw	PXD004321
67	QE_150611_131.raw	PXD004321
68	QE_150611_137.raw	PXD004321
	Escherichia coli	
69	QE_150611_140.raw	PXD004321
70	QE_150611_143.raw	PXD004321
71	QE_150611_146.raw	PXD004321
	Pseudomonas aeruginosa	
72	QE_150611_152.raw	PXD004321
73	QE_150611_155.raw	PXD004321
74	QE_150611_158.raw	PXD004321
	Moraxella catarrhalis	
75	QE_160603_81.raw	PXD004321
76	QE_160603_84.raw	PXD004321
77	QE_160603_87.raw	PXD004321
	Haemophilus influenzae	
78	QE_160627_44.raw	PXD004321
79	QE_160627_50.raw	PXD004321
80	QE_160627_52.raw	PXD004321

Table S3: The list of LC-MS/MS data files DF's downloaded from the ProteomeXchange at http://www.proteomexchange.org/ and from PeptideAtlas at http://www.peptideatlas.org/.

In house generated MS/MS data. Upload data to ProteomeXchange					
DF	*S. pneumoniae*	*S. aureus*	*E. coli*	*P. aeruginosa*	Data File Name
81	50%	50%	0%	0%	1_1ug.mzML
82	50%	50%	0%	0%	2_1ug.mzML
83	50%	50%	0%	0%	3_1ug.mzML
84	85%	15%	0%	0%	1.mzML
85	92%	8%	0%	0%	3.mzML
86	0%	0%	25%	75%	4_1ug.mzML
87	0%	0%	25%	75%	5_1ug.mzML
88	0%	0%	25%	75%	6_1ug.mzML
89	0%	0%	23%	77%	Mar01_3.mzML
90	0%	20%	0%	80%	Feb08_1.mzML
91	0%	69%	0%	31%	Feb08_3.mzML
92	0%	21%	79%	0%	Feb24_1.mzML
93	0%	70%	30%	0%	Feb24_6.mzML
94	11%	40%	18%	31%	QC4_1.mzML
95	5%	71%	9%	15%	QC4_13.mzML
96	5%	71%	9%	15%	QC4_14.mzML
97	1%	92%	2%	4%	QC4_16.mzML
98	1%	92%	2%	4%	QC4_17.mzML
99	1%	92%	2%	4%	QC4_18.mzML
MS/MS data PXD004321 downloaded from ProteomeXchange [2]					
DF	*S. pneumoniae*	*S. aureus*	*E. coli*	*P. aeruginosa*	Data File Name
100	25%	25%	25%	25%	QE_150611_161.raw
101	25%	25%	25%	25%	QE_150611_164.raw
102	25%	25%	25%	25%	QE_150611_169.raw
103	11.11%	44.44%	22.22%	22.22%	QE_160819_05.raw
104	11.11%	44.44%	22.22%	22.22%	QE_160819_08.raw
105	11.11%	44.44%	22.22%	22.22%	QE_160819_11.raw
106	44.44%	11.11%	22.22%	22.22%	QE_160819_14.raw
107	44.44%	11.11%	22.22%	22.22%	QE_160819_17.raw
108	44.44%	11.11%	22.22%	22.22%	QE_160819_20.raw
MS/MS data PXD004321 downloaded from ProteomeXchange [2]					
DF	*S. pneumoniae*	*S. aureus*	*H. influenzae*	*M. catarrhalis*	Data File Name
109	25%	25%	25%	25%	QE_160819_44.raw
110	25%	25%	25%	25%	QE_160819_47.raw
111	25%	25%	25%	25%	QE_160819_50.raw
112	44.44%	11.11%	22.22%	22.22%	QE_160819_32.raw
113	44.44%	11.11%	22.22%	22.22%	QE_160819_35.raw
114	44.44%	11.11%	22.22%	22.22%	QE_160819_38.raw
115	11.11%	44.44%	22.22%	22.22%	QE_160819_23.raw
116	11.11%	44.44%	22.22%	22.22%	QE_160819_26.raw
117	11.11%	44.44%	22.22%	22.22%	QE_160819_29.raw
MS/MS data PASS00355 downloaded from Peptide Atlas [3]					
DF	*E. faecalis*	*P. multocida*	*E. coli*	*L. acidophilus*	Data File Name
118	98.02%	0.9802%	0.9802%	0.009802%	4MUM_Run_1.raw
119	98.02%	0.9802%	0.9802%	0.009802%	4MUM_Run_2.raw
MS/MS data PASS00355 and PASS00194 downloaded from Peptide Atlas [3] 9 organisms mixture					
DF	Data File Name		9 Organisms Name		
120	9MM_Run_1.raw		*Escherichia coli*		
121	9MM_Run_2.raw		*Pasteurella multocida*		
122	9MM_FASP.raw		*Brevibacillus laterosporus*		
123	9MM_PPID.raw		*Lactobacillus acidophilus*		
			Lactobacillus casei		
			Enterococcus faecalis		
			Pediococcus pentosaceus		
			Rhodotorula glutinis		
			Saccharomyces cerevisiae		

Table S4: The list of LC-MS/MS data files (DF's) downloaded from the ProteomeXchange at http://www.proteomexchange.org/.

Human Fecal Gut Microbiome Proteomics [4]		
DF	Data File Name	Sample ID
124	Human_0.raw	PXD004039
125	Human_1.raw	PXD004039
126	Human_2.raw	PXD004039
127	Human_3.raw	PXD004039
Human Fecal Gut Microbiome Proteomics [5]		
DF	Data File Name	Sample ID
128	081915_H3_velos1_FTMS_MS2_hcd35_01.raw	PXD003907
128	081915_H3_velos1_FTMS_MS2_hcd35_02.raw	PXD003907
128	081915_H3_velos1_FTMS_MS2_hcd35_03.raw	PXD003907
128	081915_H3_velos1_FTMS_MS2_hcd35_04.raw	PXD003907
128	081915_H3_velos1_FTMS_MS2_hcd35_05.raw	PXD003907
128	081915_H3_velos1_FTMS_MS2_hcd35_06.raw	PXD003907
128	081915_H3_velos1_FTMS_MS2_hcd35_07.raw	PXD003907
128	081915_H3_velos1_FTMS_MS2_hcd35_08.raw	PXD003907
128	081915_H3_velos1_FTMS_MS2_hcd35_09.raw	PXD003907
128	081915_H3_velos1_FTMS_MS2_hcd35_10.raw	PXD003907
128	081915_H3_velos1_FTMS_MS2_hcd35_11.raw	PXD003907
128	081915_H3_velos1_FTMS_MS2_hcd35_12.raw	PXD003907
129	090115_H3_velos1_FTMS_MS2_hcd35_01.raw	PXD003907
129	090115_H3_velos1_FTMS_MS2_hcd35_02.raw	PXD003907
129	090115_H3_velos1_FTMS_MS2_hcd35_03.raw	PXD003907
129	090115_H3_velos1_FTMS_MS2_hcd35_04.raw	PXD003907
129	090115_H3_velos1_FTMS_MS2_hcd35_05.raw	PXD003907
129	090115_H3_velos1_FTMS_MS2_hcd35_06.raw	PXD003907
129	090115_H3_velos1_FTMS_MS2_hcd35_07.raw	PXD003907
129	090115_H3_velos1_FTMS_MS2_hcd35_08.raw	PXD003907
129	090115_H3_velos1_FTMS_MS2_hcd35_09.raw	PXD003907
129	090115_H3_velos1_FTMS_MS2_hcd35_10.raw	PXD003907
129	090115_H3_velos1_FTMS_MS2_hcd35_11.raw	PXD003907
129	090115_H3_velos1_FTMS_MS2_hcd35_12.raw	PXD003907
130	090415_H3_velos1_FTMS_MS2_hcd35_01.raw	PXD003907
130	090415_H3_velos1_FTMS_MS2_hcd35_02.raw	PXD003907
130	090415_H3_velos1_FTMS_MS2_hcd35_03.raw	PXD003907
130	090415_H3_velos1_FTMS_MS2_hcd35_04.raw	PXD003907
130	090415_H3_velos1_FTMS_MS2_hcd35_05.raw	PXD003907
130	090415_H3_velos1_FTMS_MS2_hcd35_06.raw	PXD003907
130	090415_H3_velos1_FTMS_MS2_hcd35_07.raw	PXD003907
130	090415_H3_velos1_FTMS_MS2_hcd35_08.raw	PXD003907
130	090415_H3_velos1_FTMS_MS2_hcd35_09.raw	PXD003907
130	090415_H3_velos1_FTMS_MS2_hcd35_10.raw	PXD003907
130	090415_H3_velos1_FTMS_MS2_hcd35_11.raw	PXD003907
130	090415_H3_velos1_FTMS_MS2_hcd35_12.raw	PXD003907
131	082015_H4_velos1_FTMS_MS2_hcd35_01.raw	PXD003907
131	082015_H4_velos1_FTMS_MS2_hcd35_02.raw	PXD003907
131	082015_H4_velos1_FTMS_MS2_hcd35_03.raw	PXD003907
131	082015_H4_velos1_FTMS_MS2_hcd35_04.raw	PXD003907
131	082015_H4_velos1_FTMS_MS2_hcd35_05.raw	PXD003907
131	082015_H4_velos1_FTMS_MS2_hcd35_06.raw	PXD003907
131	082015_H4_velos1_FTMS_MS2_hcd35_07.raw	PXD003907
131	082015_H4_velos1_FTMS_MS2_hcd35_08.raw	PXD003907

Table S4: The list of LC-MS/MS data files (DF's) downloaded from the ProteomeXchange at http://www.proteomexchange.org/.

131	082015_H4_velos1_FTMS_MS2_hcd35_09.raw	PXD003907
131	082015_H4_velos1_FTMS_MS2_hcd35_10.raw	PXD003907
131	082015_H4_velos1_FTMS_MS2_hcd35_11.raw	PXD003907
131	082015_H4_velos1_FTMS_MS2_hcd35_12.raw	PXD003907
132	090215_H4_velos1_FTMS_MS2_hcd35_01.raw	PXD003907
132	090215_H4_velos1_FTMS_MS2_hcd35_02.raw	PXD003907
132	090215_H4_velos1_FTMS_MS2_hcd35_03.raw	PXD003907
132	090215_H4_velos1_FTMS_MS2_hcd35_04.raw	PXD003907
132	090215_H4_velos1_FTMS_MS2_hcd35_05.raw	PXD003907
132	090215_H4_velos1_FTMS_MS2_hcd35_06.raw	PXD003907
132	090215_H4_velos1_FTMS_MS2_hcd35_07.raw	PXD003907
132	090215_H4_velos1_FTMS_MS2_hcd35_08.raw	PXD003907
132	090215_H4_velos1_FTMS_MS2_hcd35_09.raw	PXD003907
132	090215_H4_velos1_FTMS_MS2_hcd35_10.raw	PXD003907
132	090215_H4_velos1_FTMS_MS2_hcd35_11.raw	PXD003907
132	090215_H4_velos1_FTMS_MS2_hcd35_12.raw	PXD003907
133	090615_H4_velos1_FTMS_MS2_hcd35_01.raw	PXD003907
133	090615_H4_velos1_FTMS_MS2_hcd35_02.raw	PXD003907
133	090615_H4_velos1_FTMS_MS2_hcd35_03.raw	PXD003907
133	090615_H4_velos1_FTMS_MS2_hcd35_04.raw	PXD003907
133	090615_H4_velos1_FTMS_MS2_hcd35_05.raw	PXD003907
133	090615_H4_velos1_FTMS_MS2_hcd35_06.raw	PXD003907
133	090615_H4_velos1_FTMS_MS2_hcd35_07.raw	PXD003907
133	090615_H4_velos1_FTMS_MS2_hcd35_08.raw	PXD003907
133	090615_H4_velos1_FTMS_MS2_hcd35_09.raw	PXD003907
133	090615_H4_velos1_FTMS_MS2_hcd35_10.raw	PXD003907
133	090615_H4_velos1_FTMS_MS2_hcd35_11.raw	PXD003907
133	090615_H4_velos1_FTMS_MS2_hcd35_12.raw	PXD003907
133	090315_H5_velos1_FTMS_MS2_hcd35_01.raw	PXD003907
134	090315_H5_velos1_FTMS_MS2_hcd35_02.raw	PXD003907
134	090315_H5_velos1_FTMS_MS2_hcd35_03.raw	PXD003907
134	090315_H5_velos1_FTMS_MS2_hcd35_04.raw	PXD003907
134	090315_H5_velos1_FTMS_MS2_hcd35_05.raw	PXD003907
134	090315_H5_velos1_FTMS_MS2_hcd35_06.raw	PXD003907
134	090315_H5_velos1_FTMS_MS2_hcd35_07.raw	PXD003907
134	090315_H5_velos1_FTMS_MS2_hcd35_08.raw	PXD003907
134	090315_H5_velos1_FTMS_MS2_hcd35_09.raw	PXD003907
134	090315_H5_velos1_FTMS_MS2_hcd35_10.raw	PXD003907
134	090315_H5_velos1_FTMS_MS2_hcd35_11.raw	PXD003907
134	090315_H5_velos1_FTMS_MS2_hcd35_12.raw	PXD003907
135	090715_H5_velos1_FTMS_MS2_hcd35_01.raw	PXD003907
135	090715_H5_velos1_FTMS_MS2_hcd35_02.raw	PXD003907
135	090715_H5_velos1_FTMS_MS2_hcd35_03.raw	PXD003907
135	090715_H5_velos1_FTMS_MS2_hcd35_04.raw	PXD003907
135	090715_H5_velos1_FTMS_MS2_hcd35_05.raw	PXD003907
135	090715_H5_velos1_FTMS_MS2_hcd35_06.raw	PXD003907
135	090715_H5_velos1_FTMS_MS2_hcd35_07.raw	PXD003907
135	090715_H5_velos1_FTMS_MS2_hcd35_08.raw	PXD003907
135	090715_H5_velos1_FTMS_MS2_hcd35_09.raw	PXD003907
135	090715_H5_velos1_FTMS_MS2_hcd35_10.raw	PXD003907
135	090715_H5_velos1_FTMS_MS2_hcd35_11.raw	PXD003907
135	090715_H5_velos1_FTMS_MS2_hcd35_12.raw	PXD003907

Table S4: The list of LC-MS/MS data files (DF's) downloaded from the ProteomeXchange at http://www.proteomexchange.org/.

136	090915_H5_velos1_FTMS_MS2_hcd35_01.raw	PXD003907
136	090915_H5_velos1_FTMS_MS2_hcd35_02.raw	PXD003907
136	090915_H5_velos1_FTMS_MS2_hcd35_03.raw	PXD003907
136	090915_H5_velos1_FTMS_MS2_hcd35_04.raw	PXD003907
136	090915_H5_velos1_FTMS_MS2_hcd35_05.raw	PXD003907
136	090915_H5_velos1_FTMS_MS2_hcd35_06.raw	PXD003907
136	090915_H5_velos1_FTMS_MS2_hcd35_07.raw	PXD003907
136	090915_H5_velos1_FTMS_MS2_hcd35_08.raw	PXD003907
136	090915_H5_velos1_FTMS_MS2_hcd35_09.raw	PXD003907
136	090915_H5_velos1_FTMS_MS2_hcd35_10.raw	PXD003907
136	090915_H5_velos1_FTMS_MS2_hcd35_11.raw	PXD003907
136	090915_H5_velos1_FTMS_MS2_hcd35_12.raw	PXD003907
Human Calu-3 Cells Infected With *Orthomyxoviridae Influenzavirus A Influenza A* (H7N9)		
DF	Data File Name	Sample ID
137	OMICS_ICL102_AH1_0hr_protein_1_087_run1_25Aug14_Falcon_14-06-04.raw	PXD002385
138	OMICS_ICL102_AH1_0hr_protein_2_042_run1_17Aug14_Falcon_14-07-05.raw	PXD002385
139	OMICS_ICL102_AH1_0hr_protein_3_012_run1_17Aug14_Falcon_14-07-05.raw	PXD002385
140	OMICS_ICL102_AH1_0hr_protein_4_111_run1_23Aug14_Falcon_14-07-07.raw	PXD002385
141	OMICS_ICL102_AH1_0hr_protein_5_077_run1_rr3_2Sep14_Falcon_14-07-06.raw	PXD002385
142	OMICS_ICL102_AH1_3hr_protein_1_082_run1_27Aug14_Falcon_14-07-06.raw	PXD002385
143	OMICS_ICL102_AH1_3hr_protein_2_068_run1_17Aug14_Falcon_14-07-06.raw	PXD002385
144	OMICS_ICL102_AH1_3hr_protein_3_032_run1_23Aug14_Falcon_14-07-07.raw	PXD002385
145	OMICS_ICL102_AH1_3hr_protein_4_120_run1_23Aug14_Falcon_14-06-04.raw	PXD002385
146	OMICS_ICL102_AH1_3hr_protein_5_001_run1_17Aug14_Falcon_14-07-05.raw	PXD002385
147	OMICS_ICL102_AH1_7hr_protein_1_047_run1_27Aug14_Falcon_14-06-04.raw	PXD002385
148	OMICS_ICL102_AH1_7hr_protein_2_109_run1_23Aug14_Falcon_14-07-07.raw	PXD002385
149	OMICS_ICL102_AH1_7hr_protein_3_071_run1_17Aug14_Falcon_14-07-06.raw	PXD002385
150	OMICS_ICL102_AH1_7hr_protein_4_023_run1_15Aug14_Falcon_14-07-07.raw	PXD002385
151	OMICS_ICL102_AH1_7hr_protein_5_017_run1_17Aug14_Falcon_14-07-05.raw	PXD002385
152	OMICS_ICL102_AH1_12hr_protein_1_076_run1_27Aug14_Falcon_14-07-06.raw	PXD002385
153	OMICS_ICL102_AH1_12hr_protein_2_054_run1_17Aug14_Falcon_14-06-04.raw	PXD002385
154	OMICS_ICL102_AH1_12hr_protein_2_054_run1_rr1_4Sep14_Falcon_14-06-04.raw	PXD002385
155	OMICS_ICL102_AH1_12hr_protein_4_013_run1_17Aug14_Falcon_14-07-05.raw	PXD002385
156	OMICS_ICL102_AH1_12hr_protein_5_031_run1_27Aug14_Falcon_14-07-06.raw	PXD002385
157	OMICS_ICL102_AH1_18hr_protein_1_090_run1_15Aug14_Falcon_14-06-04.raw	PXD002385
158	OMICS_ICL102_AH1_18hr_protein_2_016_run1_17Aug14_Falcon_14-07-07.raw	PXD002385
159	OMICS_ICL102_AH1_18hr_protein_3_108_run1_23Aug14_Falcon_14-07-05.raw	PXD002385
160	OMICS_ICL102_AH1_18hr_protein_3_108_run1_25Aug14_Falcon_14-07-05.raw	PXD002385
161	OMICS_ICL102_AH1_18hr_protein_4_043_run1_15Aug14_Falcon_14-07-05.raw	PXD002385
162	OMICS_ICL102_AH1_24hr_protein_1_056_run1_17Aug14_Falcon_14-07-07.raw	PXD002385
163	OMICS_ICL102_AH1_24hr_protein_2_008_run1_17Aug14_Falcon_14-07-05.raw	PXD002385
164	OMICS_ICL102_AH1_24hr_protein_3_021_run1_15Aug14_Falcon_14-06-04.raw	PXD002385
165	OMICS_ICL102_AH1_24hr_protein_4_083_run1_17Aug14_Falcon_14-06-04.raw	PXD002385
166	OMICS_ICL102_AH1_24hr_protein_5_119_run1_rr3_3Sep14_Falcon_14-07-06.raw	PXD002385
167	OMICS_ICL102_691_0hr_protein_1_055_run1_25Aug14_Falcon_14-07-05.raw	PXD002385
168	OMICS_ICL102_691_0hr_protein_2_066_run1_27Aug14_Falcon_14-07-07.raw	PXD002385
169	OMICS_ICL102_691_0hr_protein_3_099_run1_17Aug14_Falcon_14-07-06.raw	PXD002385
170	OMICS_ICL102_691_0hr_protein_4_024_run1_27Aug14_Falcon_14-06-04.raw	PXD002385
171	OMICS_ICL102_691_0hr_protein_5_107_run1_25Aug14_Falcon_14-07-05.raw	PXD002385
172	OMICS_ICL102_691_3hr_protein_1_057_run1_27Aug14_Falcon_14-07-05.raw	PXD002385
173	OMICS_ICL102_691_3hr_protein_2_104_run1_17Aug14_Falcon_14-06-04.raw	PXD002385
174	OMICS_ICL102_691_3hr_protein_3_028_run1_23Aug14_Falcon_14-07-06.raw	PXD002385

Table S4: The list of LC-MS/MS data files (DF's) downloaded from the ProteomeXchange at http://www.proteomexchange.org/.

175	OMICS_ICL102_691_3hr_protein_4_093_run1_27Aug14_Falcon_14-07-07.raw	PXD002385
176	OMICS_ICL102_691_3hr_protein_5_069_run1_22Aug14_Falcon_14-07-06.raw	PXD002385
177	OMICS_ICL102_691_7hr_protein_1_075_run1_17Aug14_Falcon_14-07-07.raw	PXD002385
178	OMICS_ICL102_691_7hr_protein_2_059_run1_27Aug14_Falcon_14-07-05.raw	PXD002385
179	OMICS_ICL102_691_7hr_protein_3_004_run1_17Aug14_Falcon_14-07-07.raw	PXD002385
180	OMICS_ICL102_691_7hr_protein_4_097_run1_rrC3_20Aug14_Falcon_14-07-06.raw	PXD002385
181	OMICS_ICL102_691_7hr_protein_5_038_run1_rr1_2Sep14_Falcon_14-06-04.raw	PXD002385
182	OMICS_ICL102_691_12hr_protein_1_011_run1_17Aug14_Falcon_14-07-07.raw	PXD002385
183	OMICS_ICL102_691_12hr_protein_2_052_run1_27Aug14_Falcon_14-07-06.raw	PXD002385
184	OMICS_ICL102_691_12hr_protein_3_092_run1_rrC1_20Aug14_Falcon_14-06-04.raw	PXD002385
185	OMICS_ICL102_691_12hr_protein_4_035_run1_15Aug14_Falcon_14-07-07.raw	PXD002385
186	OMICS_ICL102_691_12hr_protein_5_102_run1_rr2_2Sep14_Falcon_14-07-05.raw	PXD002385
187	OMICS_ICL102_691_18hr_protein_1_061_run1_27Aug14_Falcon_14-07-07.raw	PXD002385
188	OMICS_ICL102_691_18hr_protein_2_100_run1_17Aug14_Falcon_14-07-05.raw	PXD002385
189	OMICS_ICL102_691_18hr_protein_3_020_run1_15Aug14_Falcon_14-07-07.raw	PXD002385
190	OMICS_ICL102_691_18hr_protein_4_041_run1_23Aug14_Falcon_14-06-04.raw	PXD002385
191	OMICS_ICL102_691_18hr_protein_5_026_run1_23Aug14_Falcon_14-07-06.raw	PXD002385
192	OMICS_ICL102_691_24hr_protein_1_067_run1_27Aug14_Falcon_14-06-04.raw	PXD002385
193	OMICS_ICL102_691_24hr_protein_2_002_run1_27Aug14_Falcon_14-06-04.raw	PXD002385
194	OMICS_ICL102_691_24hr_protein_3_110_run1_25Aug14_Falcon_14-07-07.raw	PXD002385
195	OMICS_ICL102_691_24hr_protein_4_085_run1_15Aug14_Falcon_14-07-05.raw	PXD002385
196	OMICS_ICL102_691_24hr_protein_5_051_run1_15Aug14_Falcon_14-07-06.raw	PXD002385
197	OMICS_ICL102_FM_0hr_protein_1_039_run1_rrC2_20Aug14_Falcon_14-07-05.raw	PXD002385
198	OMICS_ICL102_FM_0hr_protein_2_095_run1_22Aug14_Falcon_14-07-07.raw	PXD002385
199	OMICS_ICL102_FM_0hr_protein_3_101_run1_rrC3_20Aug14_Falcon_14-07-06.raw	PXD002385
200	OMICS_ICL102_FM_0hr_protein_4_078_run1_17Aug14_Falcon_14-06-04.raw	PXD002385
201	OMICS_ICL102_FM_0hr_protein_5_045_run1_17Aug14_Falcon_14-06-04.raw	PXD002385
202	OMICS_ICL102_FM_3hr_protein_1_115_run1_17Aug14_Falcon_14-06-04.raw	PXD002385
203	OMICS_ICL102_FM_3hr_protein_2_088_run1_27Aug14_Falcon_14-07-05.raw	PXD002385
204	OMICS_ICL102_FM_3hr_protein_3_003_run1_27Aug14_Falcon_14-07-07.raw	PXD002385
205	OMICS_ICL102_FM_3hr_protein_4_072_run1_25Aug14_Falcon_14-07-06.raw	PXD002385
206	OMICS_ICL102_FM_3hr_protein_5_025_run1_27Aug14_Falcon_14-06-04.raw	PXD002385
207	OMICS_ICL102_FM_7hr_protein_1_084_run1_27Aug14_Falcon_14-06-04.raw	PXD002385
208	OMICS_ICL102_FM_7hr_protein_2_010_run1_27Aug14_Falcon_14-07-05.raw	PXD002385
209	OMICS_ICL102_FM_7hr_protein_3_105_run1_17Aug14_Falcon_14-07-06.raw	PXD002385
210	OMICS_ICL102_FM_7hr_protein_4_049_run1_17Aug14_Falcon_14-07-06.raw	PXD002385
211	OMICS_ICL102_FM_7hr_protein_5_079_run1_rrC4_20Aug14_Falcon_14-07-07.raw	PXD002385
212	OMICS_ICL102_FM_12hr_protein_1_036_run1_27Aug14_Falcon_14-07-05.raw	PXD002385
213	OMICS_ICL102_FM_12hr_protein_2_114_run1_27Aug14_Falcon_14-07-06.raw	PXD002385
214	OMICS_ICL102_FM_12hr_protein_3_096_run1_25Aug14_Falcon_14-06-04.raw	PXD002385
215	OMICS_ICL102_FM_12hr_protein_4_044_run1_17Aug14_Falcon_14-07-07.raw	PXD002385
216	OMICS_ICL102_FM_12hr_protein_5_070_run1_27Aug14_Falcon_14-07-06.raw	PXD002385
217	OMICS_ICL102_FM_18hr_protein_1_007_run1_15Aug14_Falcon_14-06-04.raw	PXD002385
218	OMICS_ICL102_FM_18hr_protein_2_037_run1_rrC1_20Aug14_Falcon_14-06-04.raw	PXD002385
219	OMICS_ICL102_FM_18hr_protein_3_060_run1_15Aug14_Falcon_14-07-07.raw	PXD002385
220	OMICS_ICL102_FM_18hr_protein_4_074_run1_17Aug14_Falcon_14-07-06.raw	PXD002385
221	OMICS_ICL102_FM_18hr_protein_5_103_run1_23Aug14_Falcon_14-07-05.raw	PXD002385
222	OMICS_ICL102_FM_24hr_protein_1_029_run1_rr4_2Sep14_Falcon_14-07-07.raw	PXD002385
223	OMICS_ICL102_FM_24hr_protein_2_050_run1_27Aug14_Falcon_14-06-04.raw	PXD002385
224	OMICS_ICL102_FM_24hr_protein_3_064_run1_17Aug14_Falcon_14-07-06.raw	PXD002385
225	OMICS_ICL102_FM_24hr_protein_4_015_run1_17Aug14_Falcon_14-07-05.raw	PXD002385
226	OMICS_ICL102_FM_24hr_protein_5_091_run1_rrC2_20Aug14_Falcon_14-07-05.raw	PXD002385

See the Excel file TableS5.xlsx.

Table S5: The Excel file TableS5.xlsx contains the names and taxonomic identifiers of organisms included in DB-1 and DB-2. Organisms included in the databases were downloaded from the National Center for Biotechnology Information (https://www.ncbi.nlm.nih.gov/) on February 16, 2018.

sample source →	Identification of Influenza A (DF's 137-226)								
	Wild Type H1A			Mutant 691			Mutant FM		
Time (h)	E[R]	IF	E[CS]	E[R]	IF	E[CS]	E[R]	IF	E[CS]
0	0	0/5	0	0	0/5	0	0	0/5	0
3	0	0/5	0	0	0/5	0	0	0/5	0
7	0.00	0/5	0	1.00	2/5	1.00	0	0/5	0
12	1.00	5/5	1.00	1.00	5/5	1.00	1.00	5/5	1.00
18	1.00	5/5	1.00	1.00	5/5	1.00	1.00	5/5	1.00
24	1.00	5/5	1.00	1.00	5/5	1.00	1.00	5/5	1.00

Table S6: MiCId's viral identification results from querying DB-1 with DF's 137-226. MS/MS DF's 137-226 are from samples of Calu-3 human lung cancer cells infected with Influenza A virus (H7N9). Five technical replicates were obtained at 0, 3, 7, 12, 18 and 24 hours post infection and subjected to MS/MS analysis. The table headings are explained below: E[R] is the taxon's average rank in the identified cluster containing it; E[CS] represents the average cluster size containing the taxon; IF records the identification fraction, the numerator of which documents the number of time the known virus is identified as the head of the cluster while the denominator of which records the number of samples containing that virus.

See the Excel file TableS7.xlsx.

Table S7: The Excel file TableS7.xlsx contains the list of peptides, genus, species and proteins from the following data files (DFs) and databases (DB): DF-124 querying DB-3 (DB-Human_0_RF_6GB.fasta) and DB-4 (DB-Human_0_CF_6GB.fasta); DFs 125-127 querying DB-5 (DB-Human_1+2+3_RF_6GB.fasta); DF's 128-136 querying DB-2. These list of peptides, genus, species and proteins are used to constructed the Venn diagrams displayed in Figures 4, 5, and S3. DB-3 and DB-5 (with 'RF' in its full DB name) are based on the metagenomic reads processed by FragGeneScan [6]; DB-4 (with 'CF' in its full DB name) is based on metagenomic assembled contigs by FragGeneScan.

Taxa Assignment for Data files 100−108									
		Species				Genus			
DF	NPU	TP_f	TP_u	FP_f	FP_u	TP_f	TP_u	FP_f	FP_u
100	1017	4	4	21	21	4	4	20	591
101	1108	4	4	26	26	4	4	24	634
102	1072	4	4	23	23	4	4	24	595
103	2802	3	4	4	57	3	4	2	862
104	3001	3	4	3	70	3	4	2	858
105	3163	4	4	6	74	4	4	1	870
106	3024	4	4	66	66	4	4	2	880
107	3147	4	4	75	75	3	4	4	892
108	2598	4	4	51	51	4	4	5	866

Table S8: Species and genus identification using Unipept. DF-100 to DF-108, sample mixtures composed of four bacteria *S. pneumoniae*, *S. aureus*, *E. coli* and *P. aeruginosa*, are used to query DB-1. The number of peptides used (NPU) as input is controlled at 1% PFD using MiCId's statistics. The subscript u (or f) of the number of true positive (TP) and the number of false positive (FP) means the filtering strategy [1]) is turned off (or on).

References

1. Tanca, A., Palomba, A., Deligios, M., Cubeddu, T., Fraumene, C., Biosa, G., Pagnozzi, D., Addis, M.F., Uzzau, S.: Evaluating the impact of different sequence databases on metaproteome analysis: insights from a lab-assembled microbial mixture. PLoS ONE **8**(12), e82,981 (2013)
2. Boulund, F., Karlsson, R., Gonzales-Siles, L., Johnning, A., Karami, N., Al-Bayati, O., Ahren, C., Moore, E.R.B., Kristiansson, E.: TCUP: Typing and characterization of bacteria using bottom-up tandem mass spectrometry proteomics. Mol. Cell Proteomics (2017)
3. Tanca, A., Palomba, A., Pisanu, S., Deligios, M., Fraumene, C., Manghina, V., Pagnozzi, D., Addis, M.F., Uzzau, S.: A straightforward and efficient analytical pipeline for metaproteome characterization. Microbiome **2**(1), 49 (2014)
4. Tanca, A., Palomba, A., Fraumene, C., Pagnozzi, D., Manghina, V., Deligios, M., Muth, T., Rapp, E., Martens, L., Addis, M.F., Uzzau, S.: The impact of sequence database choice on metaproteomic results in gut microbiota studies. Microbiome **4**(1), 51 (2016)
5. Chatterjee, S., Stupp, G.S., Park, S.K., Ducom, J.C., Yates, J.R., Su, A.I., Wolan, D.W.: A comprehensive and scalable database search system for metaproteomics. BMC Genomics **17**(1), 642 (2016)
6. Rho, M., Tang, H., Ye, Y.: FragGeneScan: predicting genes in short and error-prone reads. Nucleic Acids Res. **38**(20), e191 (2010)
